# Advancements in Passive Wireless Sensing Systems in Monitoring Harsh Environment and Healthcare Applications

**DOI:** 10.1007/s40820-024-01599-8

**Published:** 2025-01-09

**Authors:** Wei Yue, Yunjian Guo, Jong‐Chul Lee, Enkhzaya Ganbold, Jia-Kang Wu, Yang Li, Cong Wang, Hyun Soo Kim, Young-Kee Shin, Jun-Ge Liang, Eun-Seong Kim, Nam-Young Kim

**Affiliations:** 1 RFIC Bio Centre, Kwangwoon University, Seoul, 01897 South Korea; 2 Department of Electronic Convergence Engineering, Kwangwoon University, Seoul, 01897 South Korea; 3 Department of Electronic Engineering, Jiangnan University, Wuxi, 214122 People’s Republic of China; 4 School of Microelectronics, Shandong University, Jinan, 250101 People’s Republic of China; 5 School of Electronics and Information Engineering, Harbin Institute of Technology, Harbin, 150001 People’s Republic of China; 6 Department of Electronics Engineering, Kwangwoon University, Seoul, 01897 South Korea; 7 Laboratory of Molecular Pathology and Cancer Genomics, Department of Molecular Medicine and Biopharmaceutical Sciences, Graduate School of Convergence Science and Technology, Seoul National University, Seoul, 08826 South Korea

**Keywords:** Wireless sensing, Passive detection, Harsh environment, Biomedical monitoring, Flexible sensors

## Abstract

Recent advancements in passive wireless sensor technology have significantly extended the application scope of sensing, particularly in challenging environments for monitoring industry and healthcare applications. These systems are equipped with battery-free operation, wireless connectivity, and are designed to be both miniaturized and lightweight. Such features enable the safe, real-time monitoring of industrial environments and support high-precision physiological measurements in confined internal body spaces and on wearable epidermal devices. Despite the exploration into diverse application environments, the development of a systematic and comprehensive research framework for system architecture remains elusive, which hampers further optimization of these systems. This review, therefore, begins with an examination of application scenarios, progresses to evaluate current system architectures, and discusses the function of each component—specifically, the passive sensor module, the wireless communication model, and the readout module—within the context of key implementations in target sensing systems. Furthermore, we present case studies that demonstrate the feasibility of proposed classified components for sensing scenarios, derived from this systematic approach. By outlining a research trajectory for the application of passive wireless systems in sensing technologies, this paper aims to establish a foundation for more advanced, user-friendly applications.

## Highlights

- This review comprehensively examines recent advancements in passive wireless systems applied to industrial environments and biomedical sensing, with a particular focus on the design strategies of passive wireless systems.
- The design principles and operational mechanisms of passive wireless system components (sensing modules and readout modules) are systematically categorized.
- Based on the latest research, the review highlights the innovative applications of passive wireless concepts in industrial environments, equipment safety, as well as in vivo and surface signal detection.

## Graphical abstract

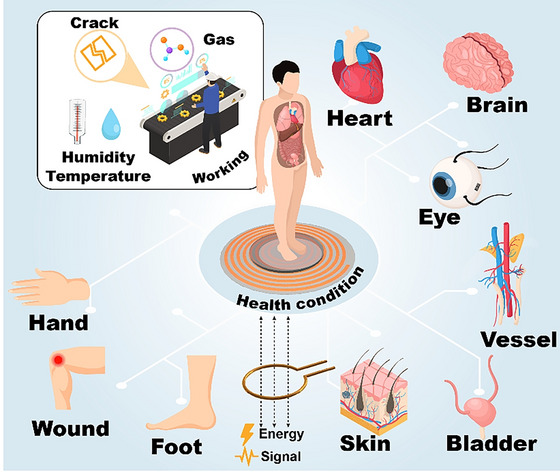

## Introduction

Advances in materials science, microelectronics, and data processing capabilities are continually propelling the evolution of smart sensing technology [1–4]. The trend involves a significant miniaturization of sensors, making them lighter and more portable while simultaneously enhancing their sensitivity, accuracy, and connectivity [5, 6]. These sensors play a crucial role in various applications, ranging from human health and pharmaceutical diagnostics to complex systems in food safety, industrial automation, and environmental monitoring [7–10]. Their capacity to convert real-world variables into quantifiable data makes them indispensable for navigating the complexities of contemporary life [11]. At the forefront of these advancements, the emergence of passive wireless sensing systems has revolutionized data acquisition and interaction modes [12–14], which are notable for their independence from onboard batteries, ease of deployment, wireless connectivity, and ability to perform without active devices [15]. Prominent passive wireless technologies, such as surface acoustic wave (SAW) sensing, self-powered sensors, and electromagnetic coupling sensors, have expanded the horizons for diverse application scenarios [16–18]. These advancements facilitate monitoring in previously inaccessible enclosed environments and enable iterative optimization of sensor deployment and performance in extreme conditions [19–21].

However, these technologies also exhibit certain limitations in specific applications. For instance, surface SAW sensors can be susceptible to mechanical vibrations and temperature fluctuations in complex environments [22, 23]. Similarly, self-powered sensors rely on a stable supply of environmental energy, making their performance vulnerable under conditions where energy harvesting is restricted [24]. In contrast, inductor–capacitor (LC) electromagnetic coupling-based passive wireless sensing systems are garnering significant attention due to their simple structure, flexible design, broad operating range, compact size, and ease of deployment. These advantageous features have enabled widespread application in the detection of various parameters, including pressure, biomarkers, temperature, and humidity [25–30]. These facile systems allow for effortless deployment and seamless integration into various environments, enabling efficient information acquisition and energy interaction using near-field coupling to provide real-time data monitoring and analysis [31]. This developed technology has significant potential applications, including deployment in extreme environments to function efficiently for monitoring chemical molecules under harsh conditions [32], functioning within the human body to provide real-time physiological states [33], and integration into lightweight wearable devices on human skin to collect epidermal data [34].

The ideal LC passive sensing system currently consists of two components: a passive sensor module for sample-specific sensing and testing, and a readout module for wirelessly reading and analyzing the resultant signals [35]. Wireless transmission methods of sensing systems have evolved from various structures, endowing these systems with the potential to perform multiple sensing tasks [36]. Implementing the system significantly depends on its alignment with the requirements of the sensing scenario, such as biocompatibility, transmission distance, and immunity to interference [37, 38]. The customized design of each aspect of the system is crucial for the effective deployment of passive sensors for optimal monitoring [39]. Understanding the interplay between each component and system construction toward the target scenario enables the optimal system configuration [40]. Some existing review articles have summarized novel applications of this system, while the construction route of scenario target sensing system components remains unclear [41]. To bridge the gap, our article offers a detailed exploration of the scenario and system interplay, focusing on the construction of components that are critical to realizing practical applications.

**Fig. 1:**
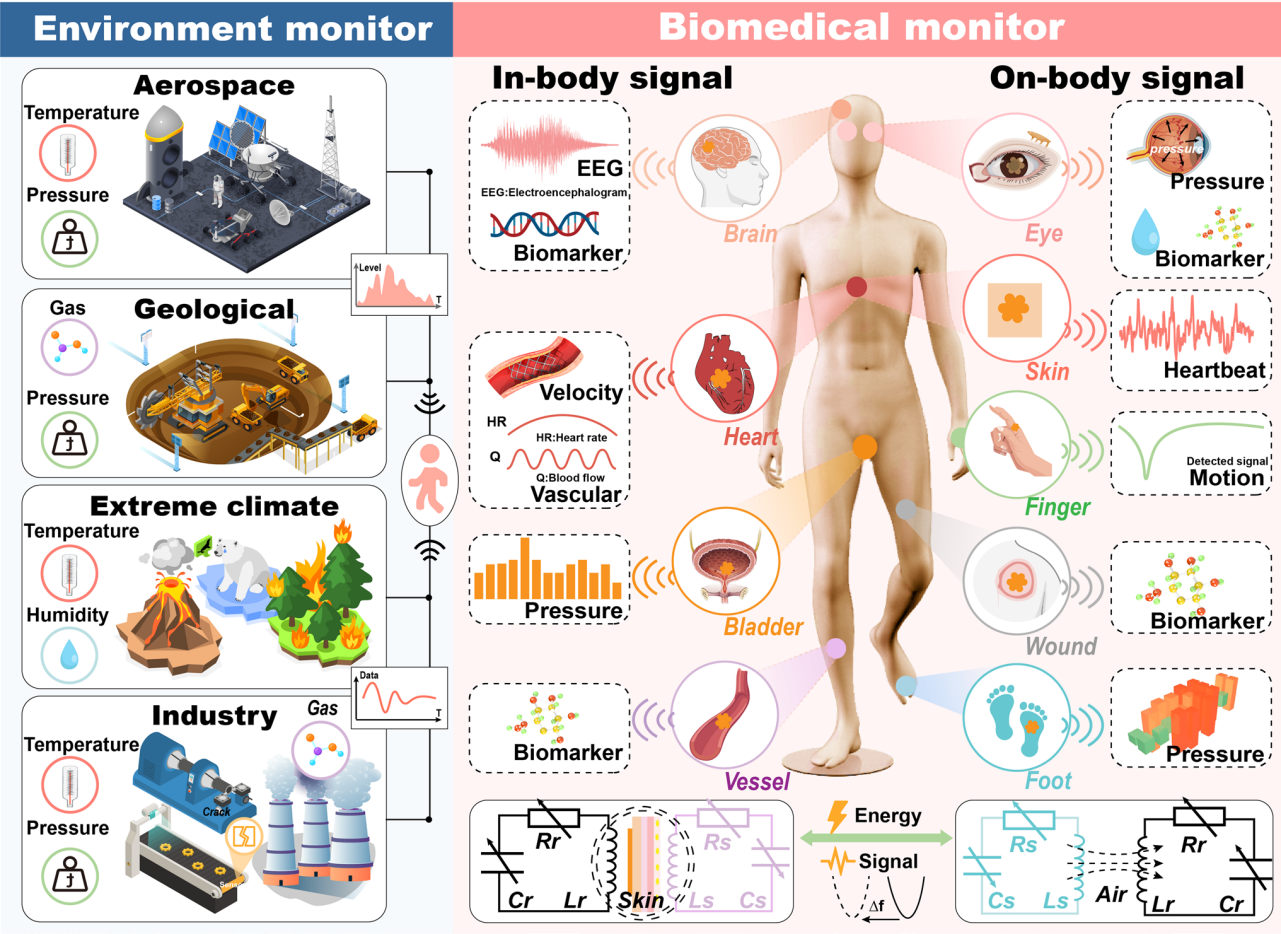
Passive wireless system for monitoring harsh environment and biomedical applications

In this review, we elucidate the detection requirements of passive wireless sensing scenarios and the corresponding design and construction routes for sensing systems [42]. It focuses mainly on categorizing and elaborating the structure and design of passive sensing and readout modules. Moreover, this review examines various practical applications for chemical and biomedical monitoring in harsh environmental scenarios and biomedical contexts, highlighting recent engineering strategies and device performance in harsh environments (such as aerospace, geological, extreme climate, and industry monitoring), internal implantable devices, and wearable epidermal sensing devices, as illustrated in Fig. 1 [43].

Specifically, passive wireless systems are crucial for monitoring various parameters in harsh environments, such as high temperatures, corrosive conditions, and chemical exposure. These systems prove valuable in diverse applications, including high-temperature and pressure monitoring in aerospace, gas concentration, and pressure measurement in geological and mining environments, temperature and humidity tracking in extreme conditions, and environmental and equipment monitoring in industrial settings [44]. Additionally, these systems offer significant advantages in health monitoring due to their flexible, multipoint deployment capabilities. For instance, implantable sensors can monitor electroencephalogram (EEG) signals and biomarkers in the brain, measure vascular flow rate and cardiac parameters, detect bladder pressure to identify blockages, and assess blood composition within vessels [45]. On the body surface, sensors can monitor intraocular pressure and tear composition, track heart rate through the skin, capture motion data from fingers, provide wound healing indicators based on exudate analysis, and assess foot pressure distribution to offer valuable health insights [46, 47].

## Passive Wireless LC Coupling Sensing Systems

In this section, sensing scenarios are discussed along with their respective types and essential requirements for deployed sensors. Subsequently, we describe the design and construction of wireless systems, providing specific design requirements and pathways for individual sensing systems. Additionally, we explore the principle of wireless transmission and the core of the system, discussing extensively the basic transmission model structure along with various variant structures for enhancement as well as parallel detection.

### Wireless System Design and Construction

**Fig. 2:**
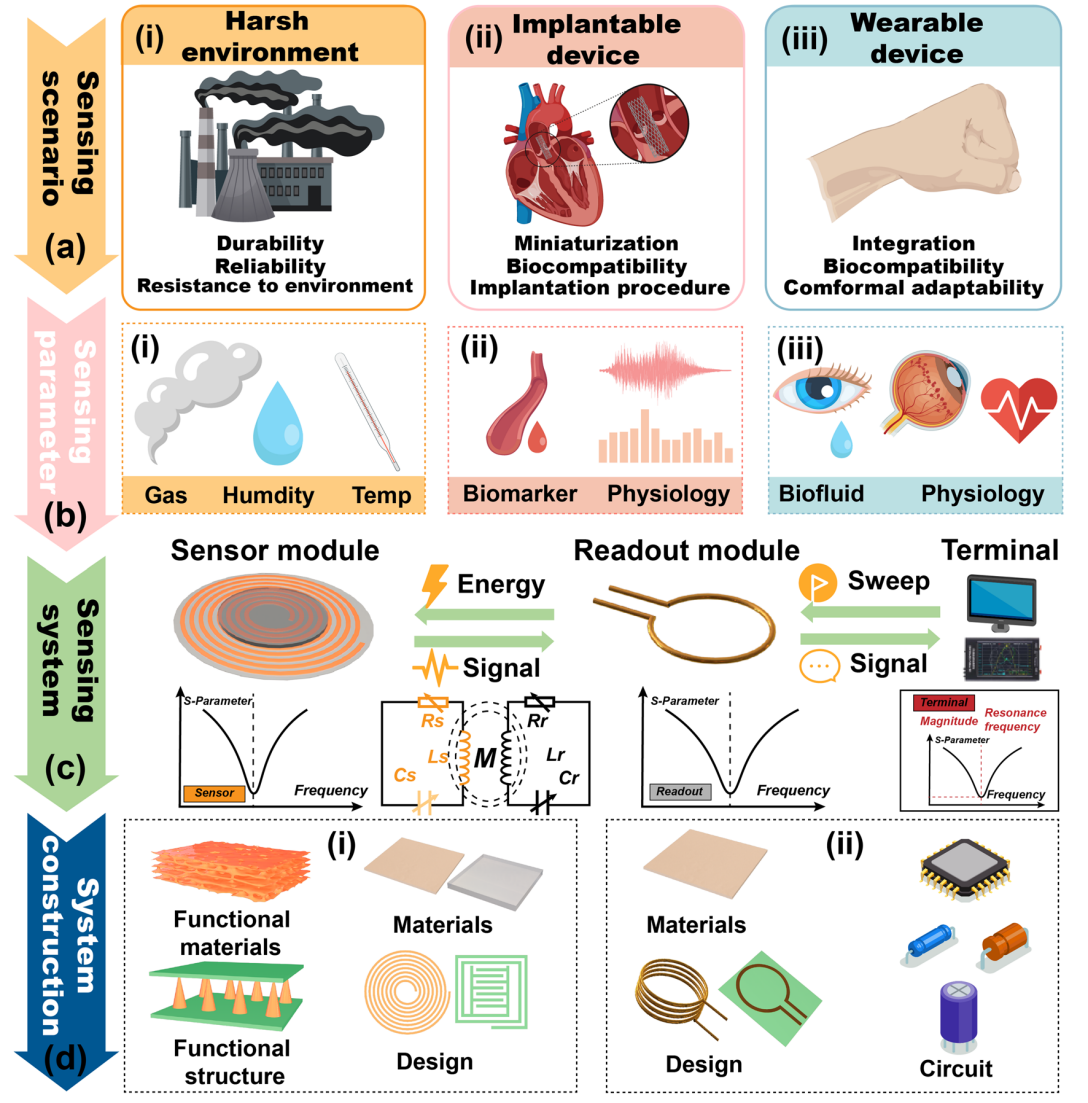
Wireless system construction route. **a** Sensing scenario: (i) harsh environment, (ii) implantable device, (iii) wearable device. **b** Sensing parameters: (i) (ii) (iii) corresponding to the above scenarios. **c** Sensing system components and **d** their considerations: (i) passive sensor, (ii) readout module

The LC-coupled wireless sensing system utilizes magnetic coupling for wireless communication, addresses layout challenges encountered in wired scenarios, and efficiently eliminates the need for power [47]. The complex design of the system, tailored for diverse applications, highlights the critical importance of aligning with the requirements of detection scenarios and design principles to optimize overall performance, as shown in Fig. 2 [48].

#### Sensing Scenario Considerations

Current designs incorporate simplified sensing components, a development stemming from advances in LC coupling system technology. This breakthrough has significantly reduced component sizes, enabling the creation of battery-free passive components [49]. Such enhancements substantially increase the system's service life, offering particular benefits in numerous critical scenarios:

Parameter monitoring in harsh environments.

Passive wireless sensors deployed in harsh environments enable real-time environmental condition monitoring such as high temperatures, corrosive conditions, and chemical exposure, thus reducing the risks associated with battery replacement (Fig. 2ai) [50, 51]. These sensors are specifically designed to endure extreme conditions, utilizing high-temperature-resistant ceramic substrates, and temperature-stable tin-doped indium oxide (ITO) electrodes. They provide a broad detection range (using modified nanomaterials) and incorporate wireless functionality and environmental resilience to ensure high durability and reliability [21, 23, 32]. In this scenario, the monitoring parameters include temperature, humidity, gas level, and pressure, and these parameters are vital for the safety of both humans and the equipment (Fig. 2ai) [52, 53].

Biomedical implants.

The practicality of passive wireless systems extends to biomedical applications, facilitating health-level detection in closed environments within the body (Fig. 2aii) [54, 55]. Such passive sensors require miniaturization and biocompatibility to minimize interference with human body functions, and an implantation procedure must be designed to simplify the surgical process. In this context, internal health indicators such as body fluid biomarker levels and internal physiological parameters such as pressure, as well as blood vessel blockage, can be monitored in real-time (Fig. 2bii), heralding a new era in personalized healthcare monitoring [27, 56].

Wearable devices for human health monitoring.

For noninvasive health monitoring, properties such as battery-free operation, user-friendliness, and affordability make passive wireless systems integral to a fully wireless and lightweight solution (Fig. 2aiii) [57, 58]. These wearable sensors are designed with a focus on flexibility and comfort, aligning with the daily activities of users. The system adeptly tracks physiological signals like heart rate and pulse and analyzes body surface fluids such as tears and sweat (Fig. 2biii) [59, 60].

The diverse applications of LC coupling systems have revolutionized their versatility and underscored their potential to transform environmental and health monitoring technologies.

#### Sensing System Considerations

The sensing system typically comprises a passive sensor module and a readout unit for magnetic coupling and power connection, characterized by scattering parameters (S-parameters) that can be measured by vector network analyzer (VNA), as illustrated in Fig. 2c. Sensing executed by passive LC sensors involves a resonant loop formed by a capacitor and a spiral inductor, which is influenced by the substance being measured, leading to frequency shifts and return loss variations in S-parameters [61]. The readout unit, connected to the power source and magnetically coupled with the inductor of the sensor (*M* represents mutual inductance coefficient), monitors the variation in the sensing resonator’s S-parameter, reflecting changes through the reflection loss and other S-parameters [62, 63]. The terminal further monitors and displays these variations, characterizing the real-time substance being measured levels.

In engineering such sensing systems, a comprehensive systematical approach is adopted, encompassing each part from passive sensors and wireless transmission methods to the customization of readout units (Fig. 2d). Passive sensors carry out sensing functions through specific components such as functional materials and structures, as illustrated in Fig. 2di [64]. The choice of electrode materials and the structural design are crucial to sensing performance and should be focused on resilience in harsh environmental conditions, biocompatibility for medical applications, and flexibility for wearable technology. The design is tailored to suit the intended applications. Wireless coupling to the reader unit, as illustrated in Fig. 2dii, and magnetic coupling play pivotal roles in the transmission of wireless energy. This process channels energy from the readout coil to the passive sensor, facilitating the feedback of signals back to the coil. The effectiveness of this magnetic coupling is closely linked to its sensing capabilities, which are defined by parameters such as transmission distance and sensitivity. When considering the readout unit (Fig. 2diii), the choice of electrode materials, pattern configuration, and the architecture of the integrated circuit module are critical in determining wireless power transmission and coupling strength, which, in turn, significantly influence the detection capability of the passive system [65, 66].

### Wireless Transmission Principle and Structure

The architecture of near-field electromagnetic coupling systems has significantly evolved, moving from a basic individual sensor-individual readout unit configuration to a range of advanced modified structures. Notably, an intermediate relay (IR) structure has been developed to amplify wireless signal strength. Meanwhile, innovative configurations such as individual readout unit-vertical sensors and individual readout unit-parallel vertical sensors have been introduced. The former facilitates the sequential processing of diverse signal types, while the latter excels in monitoring multiple-signal streams concurrently.

#### Basic Structure

**Fig. 3:**
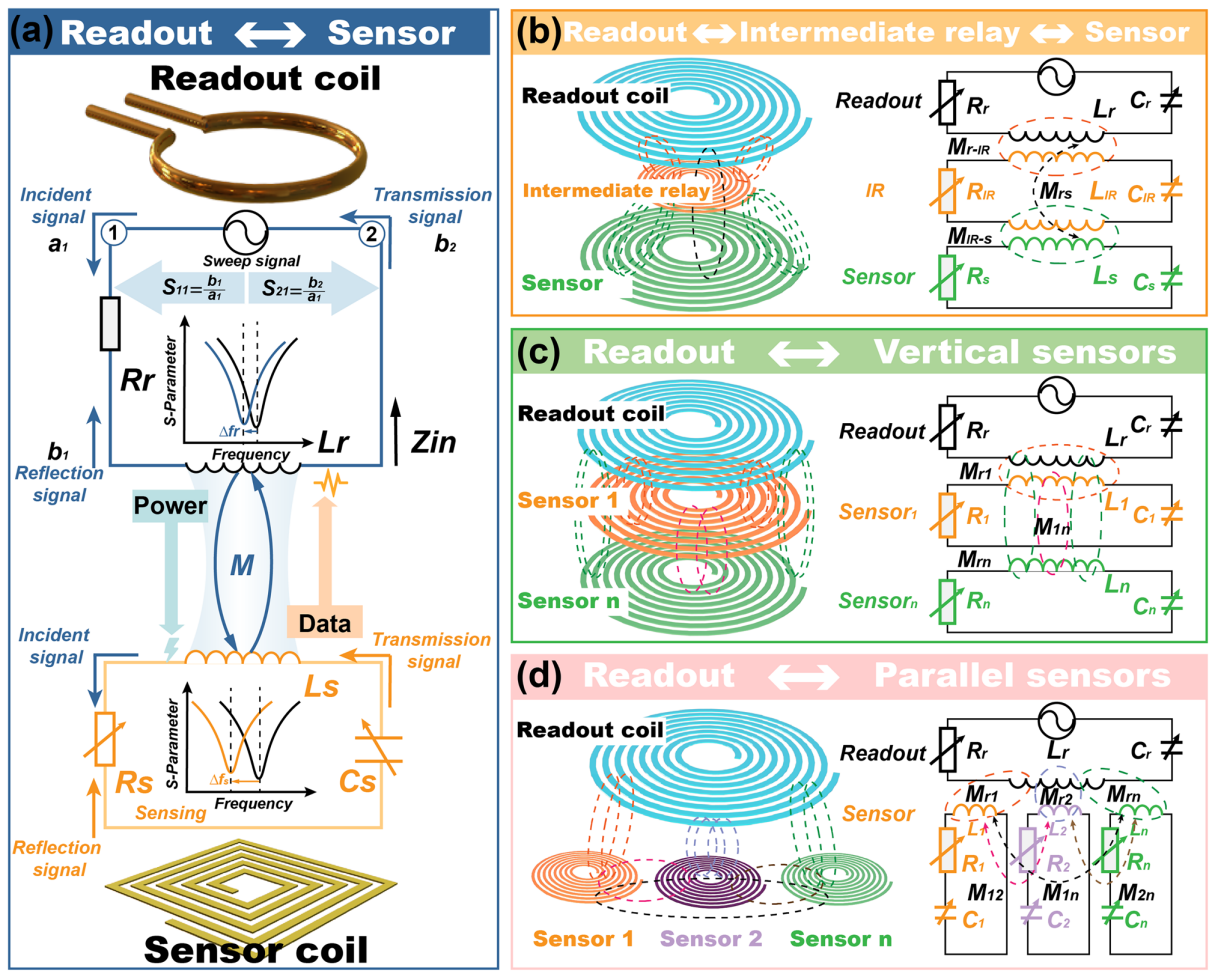
Wireless transmission structure. **a** Individual readout unit to individual sensor unit configuration. **b** Readout unit to intermediate relay and sensor configurations. **c** Readout unit to vertical sensors. **d** Readout unit to parallel sensors

The basic passive wireless LC sensor system comprises two primary components: an individual sensor unit and a readout coil connecting to the signal source (Fig. 3a) [67]. The readout coil, interfaced with the signal source, is effectively modeled by its equivalent resistance (*R*_r_) and transmitting inductance (*L*_r_), whereas subscript 'r' represents the readout coil. Conversely, the sensing unit is characterized by a combination of resistance (*R*_s_), receiving inductance (*L*_s_), and capacitance (*C*_s_), whereas subscript 's' represents the sensing unit. During the sensing process, using a two-port readout module as an example, the readout coil receives a frequency-sweep signal from the signal source and emits an electromagnetic wave signal (*a*_1_). This signal establishes wireless electromagnetic coupling with the sensor unit, interacting with it and responding to the target substance. The coupled varied signal is then reflected and transmitted back to the readout module, where the reflected wave signal (*b*_1_) and transmitted wave signal (*b*_2_) are returned to the signal source. Direct measurement of wave signals is challenging; therefore, S-parameters, as frequency-domain parameters describing the signal transmission and reflection characteristics of the sensing system, are defined as S11 = *b*_1_/*a*_1_ and S12 = *b*_2_/*a*_1_ and used for characterization [68]. These S-parameters characterize the electromagnetic signal strength of each component, reflecting the resonance frequency and signal strength. As a result, changes in the sensor unit’s S-parameters in response to the target substance lead to corresponding changes in the readout unit’s S-parameters, which are displayed on the VNA [69]. The passive sensor is designed to induce alterations in the inductor–capacitor–resistor (LCR) components toward target substance variations, thereby functioning as a sensor and resulting in changes in the reflected and transmitted power (S-parameters) and/or resonance frequency. Given that the intensity of the S-parameters is significantly influenced by environmental conditions, the inductance and capacitance components primarily govern the resonance frequency. However, because the magnetic properties of most target objects remain constant (affecting inductance *L*_s_), most resonant sensors are predominantly capacitive (affecting capacitance *C*_s_). In terms of design, a sensing element typically adopts a spiral or split-ring structure, enabling strong inductive coupling with the readout coil [70, 71]. The mutual inductance M between the readout coil and sensor can be expressed as:

1 $$M=k\sqrt{{L}_{\text{r}}{L}_{\text{s}}}$$

where *k* represents the geometry-dependent coupling coefficient, ranging from 0 (indicating no coupling) to ± 1 (indicating maximum coupling) [72]. The readout coil and sensor do not achieve magnetic resonance until their resonant frequencies align. To prevent frequency splitting—which could lead to inaccurate detection of the sensor frequency and diminished magnetic coupling—the coupling coefficient *k* between the readout coil and the sensor satisfies the following relationships:

2 $$k\le {k}_{\text{c}}=\frac{1}{\sqrt{{Q}_{\text{r}}{Q}_{\text{s}}}}$$

where *k*_c_ is the critical coupling coefficient and *Q*_r_ and *Q*_s_ are the quality factors of the readout coil and sensor, respectively [73].

3 $${Q}_{\text{r}}=\frac{1}{{R}_{\text{r}}}\sqrt{\frac{{L}_{\text{r}}}{{C}_{\text{r}}}}$$

4 $${Q}_{\text{s}}=\frac{1}{{R}_{\text{s}}}\sqrt{\frac{{L}_{\text{s}}}{{C}_{\text{s}}}}$$

when *k* = *k*_c_, the readout coil and sensor achieve a state of critical coupling, where magnetic coupling and power transmission are maximized [74]. When *k* > *k*_c_ (strong coupling), the coupling efficiency decreases and frequency splitting occurs, rendering it impossible to accurately identify the sensor frequency. Conversely, when *k* < *k*_c_, the coupling efficiency also decreases. Although the sensor frequency can be captured in this state, the signal intensity is diminished. Thus, precise detection of the sensor frequency and the strongest signal are only achieved when *k* = *k*_c_, highlighting the importance of maintaining critical coupling in the system.

#### Modified-Enhanced Structure

By incorporating an additional resonant repeater or relay, which functions both as a receiver and relay within the magnetic field, an IR coil can significantly augment magnetic coupling over extended distances, as illustrated in Fig. 3b [73, 75]. The architecture of this IR structure is a passive LC resonator meticulously tuned to a specific operating frequency [76]. In this configuration, IR structure is strategically positioned between the sensor and readout unit, establishing a sequential array of magnetically coupled resonators. This integration of IR transforms the sensing process into a repeater-node setup, where the readout unit is interconnected with a chain of magnetically coupled resonators. This setup facilitates the transfer of electromagnetic fields to subsequent nodes—passive resonator sensors—along the path length or through specifically designed inductive terminals. *M*_rs_, *M*_r-IR_, and *M*_IR-s_ are the mutual inductance between the readout coil and sensor, the readout coil and IR coil, and the IR coil and sensor, respectively.

5 $${M}_{\text{rs}}={k}_{\text{rs}}\sqrt{{L}_{\text{r}}{L}_{\text{s}}}$$

6 $${M}_{\text{r}-\text{IR}}={k}_{\text{r}-\text{IR}}\sqrt{{L}_{\text{r}}{L}_{\text{IR}}}$$

7 $${M}_{\text{IR}-\text{s}}={k}_{\text{IR}-\text{s}}\sqrt{{L}_{\text{IR}}{L}_{\text{s}}}$$

where *k*_rs_, *k*_r-IR_, and *k*_IR-s_ are the coupling coefficients between the readout coil and sensor, the readout coil and IR coil, and the IR coil and sensor [77]. *Q*_IR_ represents the quality factors of IR coil,

8 $${Q}_{\text{IR}}=\frac{1}{{R}_{\text{IR}}}\sqrt{\frac{{L}_{\text{IR}}}{{C}_{\text{IR}}}}$$

The implementation of the IR structure enhances the coupling coefficient between the readout coil and the sensor, facilitating improved signal transfer efficiency. Consequently, the sensing range achievable through magnetic coupling is expanded substantially, enabling effective signal transmission and reception over greater distances [78, 79].

In scenarios requiring the measurement of multiple sensing parameters, employing a strategy involving the vertical stacking of multiple passive sensing LC resonators can create an integrated, miniaturized multi-sensing structure, as shown in Fig. 3c [80]. This structure is interrogated by a readout coil, facilitating the miniaturization of the multi-sensing platform. The multilayer stacked architecture requires careful selection of diverse sensing materials, each chosen to ensure minimal interference among different parameters being measured. Moreover, addressing mutual crosstalk among sensing modules is a critical consideration. To mitigate this, two approaches can be adopted: designing the sensing modules to operate in distinct frequency bands and arranging the modules with specific vertical spacing and strategic horizontal positioning to minimize crosstalk. Additionally, employing decoupling algorithms is an effective method for eliminating crosstalk. This multifaceted approach to design and configuration is essential for the successful implementation of a compact and efficient multi-sensing system [81, 82].

Furthermore, in applications where miniaturization is less critical for multipoint or multiple sensing tasks, constructing a sensing matrix through the parallel stacking of multiple passive sensing LC resonators can enhance multi-sensing capabilities, as illustrated in Fig. 3d [83, 84]. In this setup, the resonant frequencies of individual sensing units are allocated to distinct frequency ranges. This arrangement ensures minimal electromagnetic crosstalk between units within the array, allowing each unit to function independently. Such a design approach is vital for the effective operation of multi-sensing systems where spatial compactness is not the primary concern, but the independence and integrity of each sensing element are paramount [85–87]. In Fig. 3c, d, mutual inductances *M*_*r1*_, *M*_*r2*_, *M*_*rn*_, *M*_*12*_, *M*_*1n*_, and *M*_*2n*_ between the readout coil and different sensors separately can be expressed as:

9 $${M}_{r1}={k}_{r1}\sqrt{{L}_{r}{L}_{1}}$$

10 $${M}_{r2}={k}_{r2}\sqrt{{L}_{r}{L}_{2}}$$

11 $${M}_{r3}={k}_{r3}\sqrt{{L}_{r}{L}_{3}}$$

12 $${M}_{12}={k}_{12}\sqrt{{L}_{1}{L}_{2}}$$

13 $${M}_{13}={k}_{13}\sqrt{{L}_{1}{L}_{3}}$$

14 $${M}_{23}={k}_{23}\sqrt{{L}_{2}{L}_{3}}$$

where *k*_*r1*_, *k*_*r2*_, *k*_*rn*_, *k*_*12*_, *k*_*1n*_, and *k*_*2n*_ are geometry-dependent coupling coefficients [88–90].

## Sensor Modules for Sensing Data

Designing a passive sensing module that fulfills the specific needs of the intended application is crucial for the development of an optimal LC-coupled wireless sensing system. Based on the configuration of the passive sensor, design modifications can be divided into three distinct types: single-stage sensor module, cascade sensor module, and specialized geometrical structures. Each sensor structure offers unique attributes that adapt the sensing capabilities of the module to meet specific application requirements in wireless sensing.

### Single-Stage Structure

**Fig. 4:**
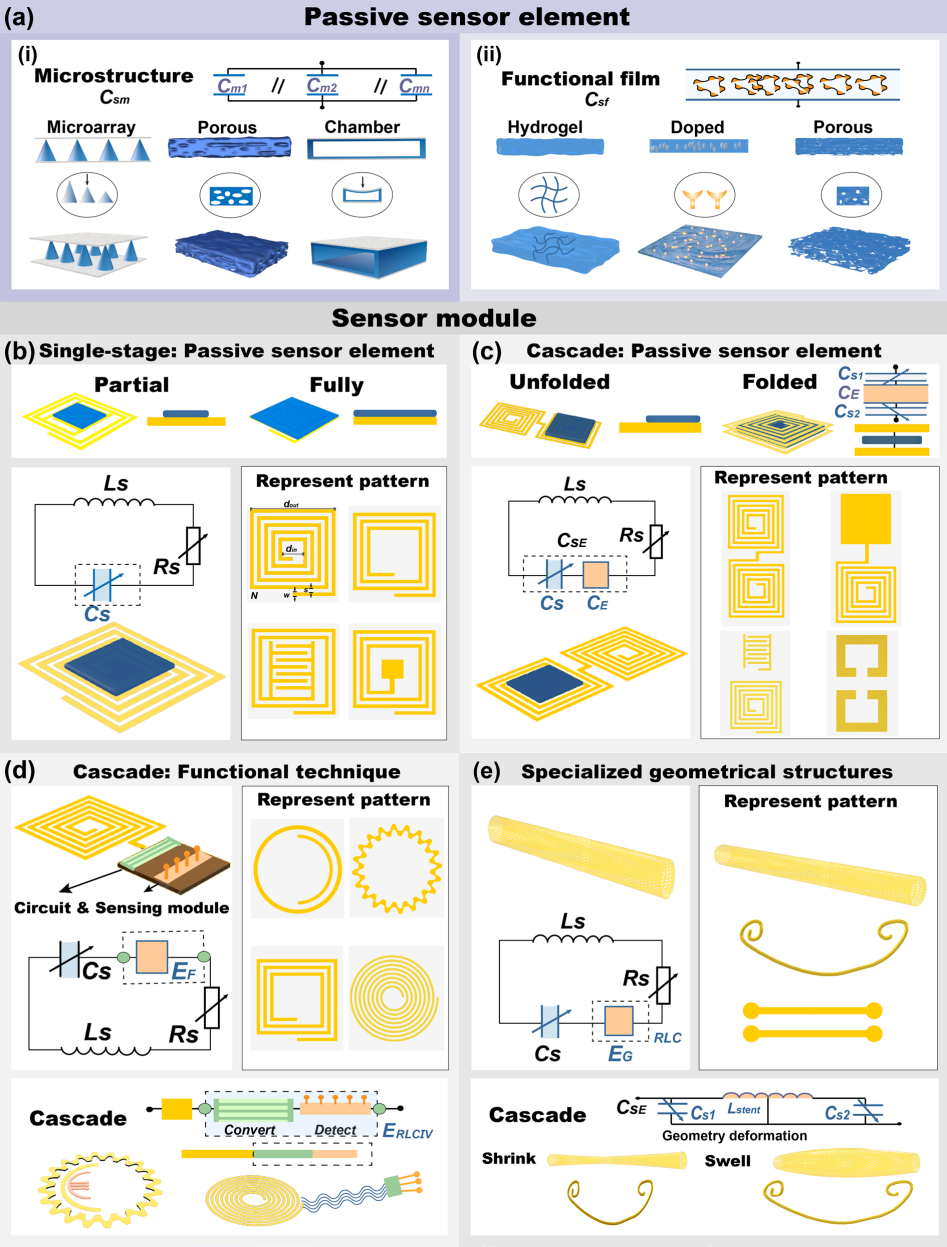
Representative structures of passive sensing modules. **a** Sensing structure: (i) microstructure, (ii) functional film. **b** Single-stage sensor module. **c** Cascade module of integrating with passive sensor element. **d** Cascade structure combined functional techniques. **e** Specialized geometrical structures

As illustrated in Fig. 4a, in a typical passive sensor module, the passive sensor element is generally positioned at the location of the sensor unit's strongest electromagnetic field to achieve a specific response to the target substance (Fig. 4b, c). Therefore, the design and selection of these structures are crucial and typically categorized into microstructures and functional films. As depicted in Fig. 4ai, commonly used microstructures include microarrays (such as pyramidal and micropillar arrays), porous structures, and cavity structures, with material selection tailored to the specific measurement target [91]. These microunit matrices (microarrays and porous structures) can be likened to an array of capacitors connected in parallel, where the capacitance of each unit depends on the target analyte, collectively influencing the overall capacitance of the sensing module (*C*_sm_). Cavity structures, analogous to parallel plate capacitors, exhibit capacitance (*C*_sm_) changes based on varying distances between the plates. Similarly, a functional film can be applied to a sensitive area (Fig. 4aii) to act as an additional circuit capacitor [92, 93]. The intrinsic material and structural features, such as hydrogels with designed internal networks, doping-sensitive nanomaterials, or micrometer-size porous structures of the functional film, are designed to respond to the target substance, resulting in a detectable change in capacitance (*C*_sf_). This change in capacitance affects the overall capacitance (*C*_s_) of the sensing module, thereby influencing its resonance characteristics such as the resonance frequency and S-parameters.

Furthermore, as illustrated in Fig. 4b, a single-stage sensor module primarily consists of passive sensor elements and spiral coil-based patterns. Representative patterns include spiral coils (characterized by parameters such as the number of turns *N*, line width *w*, and spacing *s*), as well as spiral coils interconnected with interdigitated electrodes (IDE) or planar electrodes [94–96]. The system can be modeled as a series connection of equivalent inductance (*L*_s_), resistance (*R*_s_), and capacitance (*C*_s_). The inductance is typically determined by the pattern within this equivalent circuit, which defines the inductance-capacitance resonance frequency [97, 98]. For planar spiral inductor, its inductances can be calculated by:

15 $$L={K}_{1}\mu \frac{{N}^{2}{d}_{\text{avg}}}{1+{K}_{2}\rho }$$

where *N* is the turn number, and *d*_avg_ = (*d*_in_ + *d*_out_)/2 represents the average diameter, *σ* = (*d*_out_–*d*_in_)/(*d*_in_ + _dout_) represents the fill ratio, *μ* is the permeability, *K*_1_ and *K*_2_ can be extracted from shape. This equation can be used to calculate square, hexagonal, octagonal, and circular inductors. The overall capacitance can be actively modulated by incorporating passive sensor element for responding to target substances that either partially or entirely cover the coil at the location of the strongest electromagnetic field, as illustrated in Fig. 4a, thus reflecting in S-parameter variation.

### Cascade Structure

A cascade-modified sensor consisting of passive film or integrating functional techniques is a common structure in sensing applications, as depicted in Fig. 4c, d [99, 100]. The cascade pattern structures depicted in Fig. 4c are implemented in passive sensor element-based systems. Representative patterns can be categorized into two classes: interconnected and separate configurations. The interconnected patterns include interconnected spiral coils and planar electrodes integrated with spiral coils. The separate patterns consist of individual spiral coils combined with IDE, as well as split-ring resonator (SRR) structures. The system can be modeled as a series connection of equivalent inductance, resistance, capacitance, and the electrical parameters of the sensing unit, which comprises passive sensor elements that respond to target substances. Furthermore, the direct modification approach based on passive sensor elements is classified into unfolded and folded modes [101, 102]. The sensing unit in the unfolded mode based on interconnected forms follows the modification strategy and operational mechanism described in Sect. 3.1, with cascaded unmodified helical components serially connected through a common electrode. The folded mode is based on interconnected patterns, where spiral coils are folded in half and aligned, with a dielectric layer introduced between the antenna segments to incorporate the sensing element. On the other hand, the cascade patterns are achieved by stacking separate sensor elements and sandwiching a passive sensor structure in between [103]. This design generates interlayer capacitance (*C*_E_), which significantly influences the resonance parameters of the cascaded sensing module (*C*_SE_). By leveraging the inherent characteristics of the antenna structure, this approach enables tailored responses to specific sensing requirements and enhances overall functionality.

Additionally, cascade structures integrating spiral interconnects with other functional technologies are also common, as illustrated in Fig. 4d [104, 105]. Representative patterns of such structures include spirals in various shapes (circular, square, meander, etc.), which are directly interconnected with functional technology modules. On the one hand, these spirals can receive and transfer energy from the signal source to the technology module (usually set at 13.56 MHz); on the other hand, they can receive detection data from the functional technology module and feedback this information to the signal source. Common functional modules include auxiliary technologies such as electrochemical electrodes, photoelectric arrays, and near-infrared (NIR) LED, generating electrical signals such as capacitance, resistance, voltage, and current, etc. [67, 106]. Depending on the specific sensor module (*E*_F_) configurations, these integrated systems can be classified into two main types. The first type directly influences the circuit's overall capacitance, inductance or resistance to affect its resonant parameters, where the obtained target signals—such as capacitance (*E*_C_), resistance (*E*_R_), or inductance (*E*_L_)—immediately alter the resonance characteristics (S11, frequency), facilitating wireless readout [107, 108]. The second type involves processing the detected signals, including current (*E*_I_), voltage (*E*_V_), or resistance (*E*_R_), using conversion circuits such as amplifiers, analog-to-digital converter (ADC) to accurately transform analog signals into digital signals [109]. These digital signals are subsequently stored in dedicated chips and wirelessly transmitted to a terminal through the antenna [110].

### Specialized Geometrical Structures

In addition to the aforementioned single-stage and cascade sensing modules based on spiral coils, specialized geometrical structures tailored for specific scenarios have also been developed, as shown in Fig. 4e. Representative patterns include implantable spiral stent-like structures and brackets that are suitable for in vivo applications. These structures, known for their unique flexibility and elasticity, are particularly suited for applications requiring bending and stretching, making them ideal for integration into flexible sensing modules [111]. Such structures can be modeled as a series connection with equivalent inductance (*L*_S_), resistance (*R*_S_), capacitance (*C*_SE_), and electrical parameters of the sensing unit (*E*_C_). These structures can respond to external stimuli or changes in target materials through morphological transformations such as stretching, contraction, bending, and torsional deformation, resulting in variations in capacitance (*C*_S_) and inductance (*L*_S_). Additionally, they can integrate ultra-small passive sensor modules on the scaffold to achieve specific sensing functions, thereby inducing changes in the S-parameters [105].

## Readout Modules for Analyzing Data

**Fig. 5:**
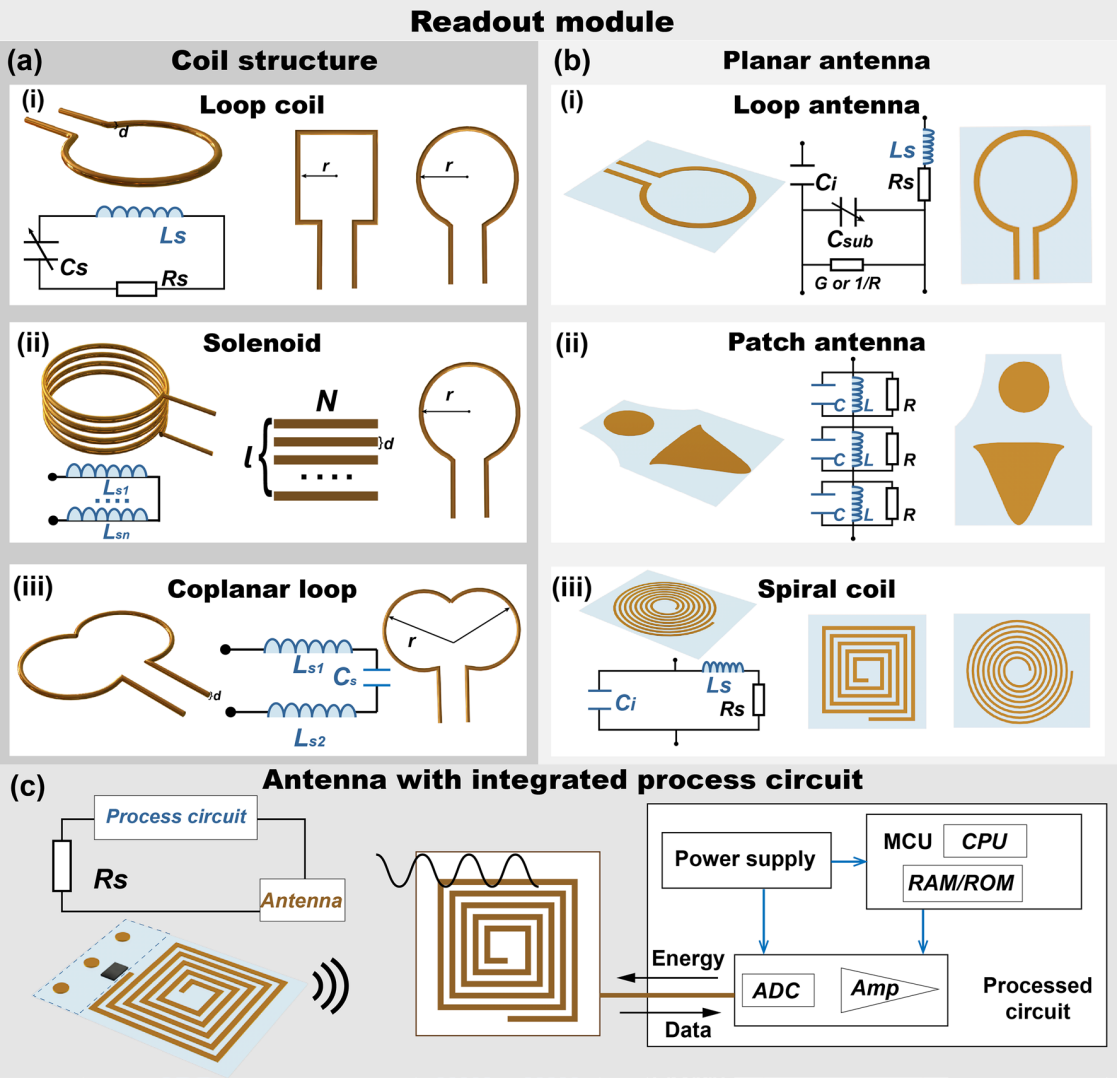
Representative structure of wireless readout module. **a** Coil structure: (i) loop coil, (ii) solenoid, (iii) coplanar loop coil. **b** Planar antenna: (i) loop antenna, (ii) patch antenna, (iii) planar spiral coil. **c** Planar-based antenna integrated with process circuit

Similarly, designing a readout module that meets the specific requirements of the application scenario for accurate acquisition of wireless sensing data is crucial in developing an optimal LC-coupled wireless sensing system. In this context, wireless readout antennas can be divided into three distinct types: coil structure, planar antennas, and planar-based antennas integrated with embedded processing circuitry. Each type is designed to meet different scenario demands, with the structure of the antenna being a critical factor in determining its suitability for various wireless sensing applications. This categorization aids in selecting an appropriate readout antenna design that aligns best with the unique requirements of the intended application, thereby enhancing the overall effectiveness of the LC-coupled wireless sensing system, as illustrated in Fig. 5.

### Loop Coils

The coil structure, typically made from metal wires, is the most common in readout module designs [35]. It is usually connected to a VNA via a radio-frequency cable, which excites the coil to exhibit specific characteristics, such as resonance frequency (Fig. 5ai) [71]. Theoretically, the coil can be represented as a combination of equivalent inductance (*L*_S_) and resistance (*R*_S_), generating a localized electromagnetic field at its center. This field wirelessly couples with the passive module, facilitating the collection of induction signals that are subsequently analyzed for data [112, 113]. The coil's material, wire diameter (*d*), number of turns, and geometry size (radius *r*) are pivotal factors that define its characteristics. Structurally, coils can be classified into three categories: single-turn loop coils, multi-turn solenoids, and coplanar loop coils. The single-turn coil, with its basic structure, allows for modulation of the resonance frequency and impedance through adjustments in coil size, and it is typically positioned concentrically with a passive module for use in various passive wireless sensing systems. Multi-turn coils, created by winding the wire multiple times, enable modulation of the resonance frequency while simultaneously enhancing the magnetic field strength and coupling efficiency, leading to more effective energy transmission and increased sensitivity (Fig. 5aii) [114, 115]. The inductance can be calculated as [72]:

16 $$L=\eta \frac{{\mu N}^{2}\pi {r}^{2}}{l}$$

where *N* and *r* represent the number of coil turns and geometry radius, respectively. *μ* is the permeability. *l* is the solenoidal length, and $\eta$=*l/r* is the dimensionless factor. These parameters are related to the overall inductance and the corresponding S-parameters. Furthermore, coplanar loop coils, which involve overlapping two circular single-turn coils, generate an intensified electromagnetic field in the overlapping region, making them suitable for enhanced sensing applications (Fig. 5aiii) [116, 117]. The wire diameter (*d*), geometry radius (*r*), and overlap area in Fig. 5aiii affect overall inductance (*L*_S_) as well as capacitance (*C*_S_).

### Planar Antennas

Planar microstrip antennas, typically connected to VNA through a single port, are stimulated to emit and receive electromagnetic waves, as depicted in Fig. 5b [87]. Their intricate design is often optimized to function most effectively at a specific frequency, namely the resonance frequency, facilitating efficient signal coupling and reception [102]. The structural parameters of these antennas can be theoretically represented by the equivalent resistance (*R*_S_), inductance (*L*_S_), and capacitance (*C*_i_) of the pattern, as well as resistance (*R*) and capacitance (*C*_sub_) to the ground. The resonance frequency is determined using computational formulas, with the bandwidth ascertained through simulations [118]. A frequency range characterized by a return loss below -10 dB is commonly regarded as the effective operational bandwidth for these antennas. Literature reviews suggest that these antennas can achieve longer working distances than traditional readout coils [41]. Among various designs, the coplanar waveguide-fed monopole antenna has been recognized for its effectiveness in readout LC sensor data (Fig. 5bi) [119, 120]. Ultra-wideband (UWB) high-gain directional antennas can be modeled as cascaded LCR networks. They are frequently used and characterized by linear polarization and significant return losses (Fig. 5bii) [121]. Additionally, the spiral planar antenna, renowned for its multimode radiation and wide operational bandwidth, is another popular design (Fig. 5biii). The number of turns (*N*), line width (*w*), and spacing (*s*) in circular and square spiral patterns are straightforward to adjust, allowing for precise control over the total inductance (*L*_S_), capacitance (*C*_i_), and tunability. These parameters not only determine the antenna's electrical characteristics but also enable multi-band reception and transmission capabilities, along with excellent directional performance [97].

### Antenna with Integrated Process Circuit

In wireless detection scenarios that demand high data accuracy and quality, meticulously engineered circuit modules for wireless collection and data processing are paramount, as illustrated in Fig. 5c [122]. These modules typically include a suite of essential components: readout coils for inductive coupling, built-in oscillators, conversion modules, amplification modules, and various other circuit elements seamlessly integrated into microcontrollers and micropower supplies [123]. Together, these components synergistically ensure reliable data acquisition and efficient data processing. The readout coil plays a crucial role, wirelessly receiving sensor data signals and facilitating energy transmission to the sensors via inductive coupling. The built-in oscillator is pivotal in generating stable clock signals and coordinating the synchronous operation of the circuit. The conversion module converts the captured analog signals into digital format, simplifying the processing workload of the microcontroller. Amplification modules boost these signals, ensuring effective data transmission over extended distances or in scenarios with attenuated signals. The microcontroller, serving as the system's brain, handles data processing, analysis, and storage, while the micropower supply provides consistent energy, underpinning the system's functionality. This holistic approach to circuit module design is indispensable in modern wireless detection systems, meeting the strict requirements for data integrity and efficiency [124].

## Applications

### Harsh Environment Monitoring

#### Environment Parameter Monitoring

**Fig. 6:**
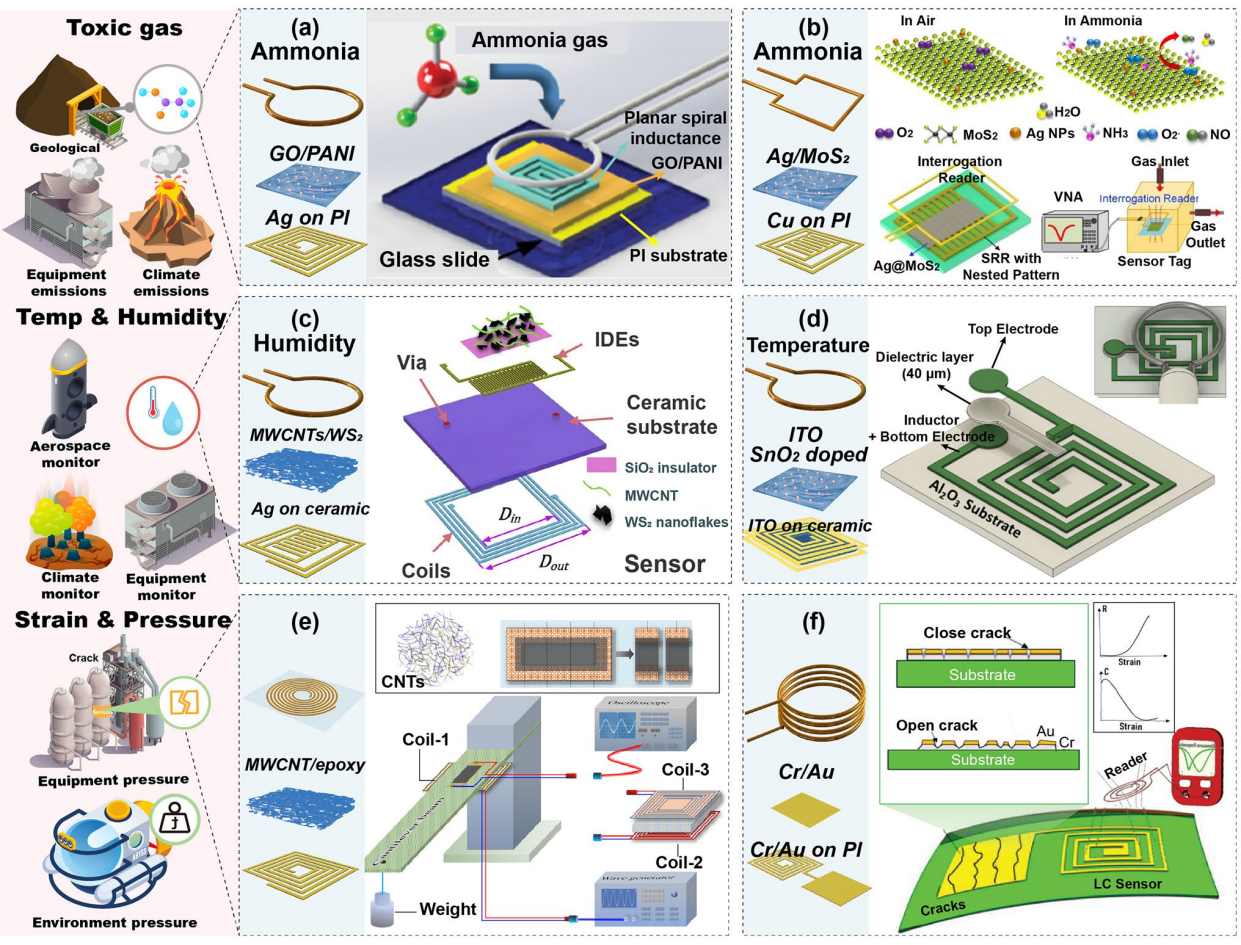
Examples of harsh environment and equipment monitoring. **a** NH_3_ sensing using GO/PANI film. Reproduced with permission from Ref. [127] Copyright © 2019 John Wiley & Sons Inc. **b** NH_3_ detection using Ag@MoS_2_ film. Reproduced with permission from Ref. [128] Copyright © 2021 Elsevier. **c** Humidity sensing using WS_2_ MWCNTs nanoflakes. Reproduced with permission from Ref. [96] Copyright © 2019 Elsevier. **d** Temperature sensing. Reproduced with permission from Ref. [23] Copyright © 2023 John Wiley & Sons Inc. **e** Equipment crack detection. Reproduced with permission from Ref [135]. Copyright © 2023 Elsevier. **f** Tank equipment crack detection. Reproduced with permission from Ref. [136] Copyright © 2023 John Wiley & Sons Inc

In harsh environments, monitoring parameters like temperature, humidity, and gas concentrations is vital for safeguarding personnel health while also ensuring the safety and efficiency of equipment operations [21]. Sensing systems need to provide real-time functional monitoring capabilities without relying on active devices, while also being robust enough to endure harsh conditions [20]. This dual focus on functionality and durability is essential to ensure that environmental conditions within harsh environments are consistently maintained within safe and optimal ranges. Gas monitoring in harsh environments across different scenarios is of critical importance [125]. For example, detecting toxic and hazardous gases such as ammonia (NH_3_) and hydrogen sulfide (H_2_S), or monitoring oxygen levels in mines, can provide early warnings of potential dangers, ensuring the safety of workers [126–128]. Real-time monitoring of equipment emissions, including carbon dioxide (CO_2_), nitrogen oxides (NO_2_), and volatile organic compounds, not only helps identify equipment malfunctions or abnormal operations but also reduces threats to ecosystems and human health. Additionally, monitoring gases during natural disasters, such as volcanic eruptions, aids in predicting volcanic activity trends and issuing timely warnings [125, 126]. Wu et al. developed an innovative passive wireless capacitive sensing system for detecting NH_3_ (Fig. 6a) [127]. The passive sensing antenna, based on an LC resonator with a spiral structure attached to a suction filter, when combined with a graphene oxide/polyaniline (GO/PANI) nanocomposite on a polyimide (PI) substrate, responds to NH_3_ concentration by altering the barrier capacitance of the composite. This change produces a shift in the resonance frequency, which is wirelessly read out by a single-loop readout coil with high sensitivity and fast response to NH_3_ concentrations ranging from 0 to 100 ppm. Similarly, Zhang et al. introduced silver nanoparticle-decorated molybdenum disulfide nanosheets (Ag@MoS_2_) on a nested SRR, exhibiting a frequency shift with increasing NH_3_ concentration (Fig. 6b) [128]. The interrogation reader wirelessly detects this variation, providing real-time NH_3_ concentrations for industrial or poultry farms. This system demonstrated high sensitivity to NH_3_ of 0.097 ppm^−1^ and a low limit of detection (LOD) of less than 1 ppm.

Environmental conditions such as humidity and temperature are critical physical parameters that significantly impact the performance of both personnel and equipment. In aerospace environments, controlling temperature and humidity is crucial for astronaut health and equipment reliability [129–131]. In industrial settings, temperature and humidity regulation affect equipment lifespan and worker safety, as abnormal levels can accelerate equipment aging or create safety risks [132]. Monitoring these parameters during extreme weather conditions helps predict and respond to environmental changes, reducing potential risks to personnel, equipment, and ecosystems [21]. For essential humidity detection, Lv et al. developed a wireless LC humidity detection system based on multi-walled carbon nanotubes (MWCNTs)/ tungsten disulfide (WS_2_) nanoflakes (Fig. 6c) [96]. The passive sensor uses a spiral inductor coil and IDE screen printing on an alumina ceramic substrate, with WS_2_ nanosheets sprayed on the IDE. This configuration offers a large surface area and active sites for interaction with gas molecules, making it an effective material for adsorbing and responding to humidity. This response causes changes in capacitance and resonance frequency, which are wirelessly read out via a single-ring readout coil, exhibiting stability, high sensitivity, and low hysteresis over a wide humidity range (10%–95%RH). For monitoring temperature, ITO is resistant to high temperatures and exhibits a sensitive conductivity change in response to temperature. Kavin et al. used ITO as a temperature-sensitive material and developed a passive wireless temperature system with ITO deposited on an aluminum oxide (Al_2_O_3_) substrate and a stacked folded dielectric layer with Al_2_O_3_ as an interlayer (Fig. 6d) [23]. The system responds to temperature with a change in capacitance, causing a shift in the resonance frequency, which is wirelessly read by a single-loop readout coil, allowing stable temperature sensing up to 1200 °C. Thus, this system is well suited for temperature detection in harsh, high-temperature environments.

#### Mechanical Parameter Monitoring

Harsh environments, such as aerospace or industrial settings, require careful monitoring of pressure and stress, as these factors are critical to ensuring safety [22]. In aerospace, fluctuations in pressure and structural stress can compromise the integrity of critical systems, potentially leading to equipment failures or safety risks for personnel [133]. Similarly, in industrial environments, continuous monitoring is essential for detecting early signs of mechanical wear, fatigue, or failure [134]. Regular wireless monitoring of these aspects can help maintain the smooth operation of plant machinery and provide timely feedback to staff, thereby protecting the workforce and the manufacturing process. Sensors deployed for machine operating condition monitoring need to be highly durable and reliable to withstand harsh environments, capable of real-time data transmission, and compatible with existing systems. Huang et al. developed a wireless strain sensor using a MWCNT/epoxy composite membrane, where the carbon nanotube network forms a complex LCR network of resistors, inductors, and capacitors to quickly respond to strain (Fig. 6e) [135]. The LCR network acts as a passive sensor that responds to strain, generating a resonance frequency shift. A helical coil connected to the VNA interacts with the film via radiomagnetic excitation to read the strain value. The high adhesive compatibility of the thin film allows easy integration and fixation on various surfaces, enabling the measurement of device breakage without complicated installation procedures or damage to the device. Nesser et al. developed a highly sensitive radio-frequency identification (RFID) wireless sensing system for structural health monitoring (Fig. 6f) [136]. The sensor element consists of a cascade of helical wires and a thin, flexible sensing component with a parallel capacitive plate that detects cracks and displays a resistive step with the degree of cracking, producing a frequency shift that the wireless readout coil can read. The sensing system can detect strains as small as 1% with a response time of less than 10 ms.

#### Integrated Multiparameter Monitoring

**Fig. 7:**
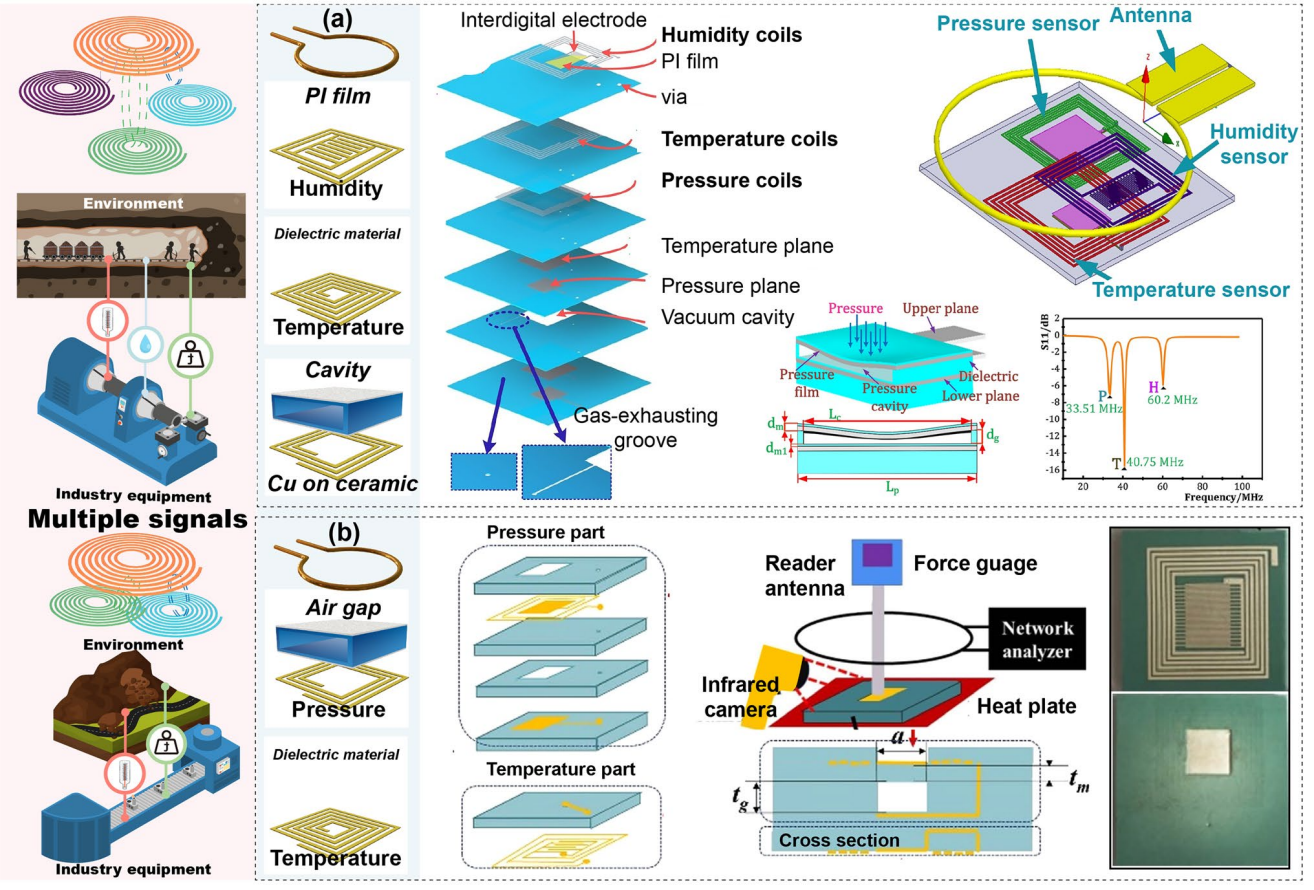
Example of factory multiple parameters monitoring. **a** TPH wireless detection system. Reproduced with permission from Ref. [137] Copyright © 2018 Elsevier. **b** Pressure and temperature detection system. Reproduced with permission from Ref. [138] Copyright © 2018 Elsevier

As mentioned earlier, individually monitoring environmental parameters such as temperature and humidity, as well as mechanical parameters such as pressure, is crucial for ensuring the safety of personnel and equipment in harsh environments. Clearly, simultaneously tracking multiple parameters—including temperature, humidity, vibrations, and structural integrity—can significantly reduce the risk of unexpected failures and provide a more comprehensive monitoring approach [21]. For example, in enclosed environments like mining tunnels or during extreme weather, monitoring multiple parameters simultaneously can provide early warnings of potential hazards to workers. Additionally, monitoring equipment helps detect abnormal conditions early, enabling timely intervention and maintenance [22]. Passive wireless systems offer a streamlined solution for integrating multiple passive sensors with a single coil through the transmission mode of readout-vertical sensors. Tan et al. proposed an LC pressure–temperature–humidity (TPH) multilayer sensor structure integrated on low-temperature co-fired ceramic (LTCC) ceramics. Helical coils with varying turns and wire widths were designed for different sensing parameters, allowing each to operate in distinct frequency bands (Fig. 7a) [137]. A pressure chamber, LTCC ceramics, and a PI membrane were designed as single-parameter sensing materials that did not interfere with one another. The multiple signals of the integrated sensor can be read out by a single coil for pressure, temperature, and humidity sensing, operating at 33, 40, and 60 MHz, and stably functioning at 25%–200 °C, 70–220 kPa, and 24%–90%RH. This broad applicability demonstrates the wide range of applications for such a system. Lin et al. developed an integrated passive wireless pressure–temperature dual-parameter sensor based on LTCC technology (Fig. 7b) [138]. The air cavity capacitor enables pressure sensing by generating capacitance variations in response to external pressure, while temperature sensing is achieved through the capacitance-inductance changes generated by the thermal expansion characteristics of the coil electrode material. Their sensing coils operate at 168 and 58 MHz frequency bands, respectively. A single readout coil can extract data from the dual-parameter sensing system, which operates within the range of 140–850 kPa and 50–500 °C. These innovations underscore the efficiency of passive wireless systems in industrial monitoring settings. Table 1 provides an overview of the diverse applications of LC passive wireless systems in harsh environments, including exposure to toxic gases, high temperatures, and extreme pressures. These systems typically feature simple sensing structures, often configured as single-stage designs in which the antenna is integrated with specific sensing film or structure. The characteristics of this component dictate the system’s operating range and sensitivity, offering advantages such as cost-effectiveness and high sensing performance.

**Table 1:** Passive wireless sensing systems for harsh environment monitoring

References	Application		Sensor module		Working frequency (MHz)	Dimension (mm)	Sensitive structure/material	Response type	Sensing range	Sensitivity	Readout module		Characteristic
	Scenario	Target	Structure	Material							Structure	Material	
[127] 6a	Harsh environment	NH_3_ gas	Single: Square spiral line + film	Ag on PI	1376	–	GO/PANI film	Capacitance	0–100 ppm	49.3 × 10^–5^ ppm	Loop coil	Cu	High sensitivity and fast response
[128] 6b	Harsh environment	NH_3_ gas	Single: Square spiral line + film	Cu on PI	1000	–	Ag/MoS_2_ film	Permittivity	0–100 ppm	0.097% / ppm	Loop coil	–	High sensitivity and low detection limit
[96] 6c	Harsh environment	Humidity	Single: Square spiral line/IDE + film	Ag on ceramic	70	32 × 32	MWCNTs/WS_2_	Permittivity	10%–95%RH	15%–55%RH: 2.201 kHz/%RH 55%–95%RH: 70.684 kHz/%RH	Loop coil	Cu	High humidity range, high repeatability and stability
[23] 6d	Harsh environment	Temperature	Single: Square spiral line/IDE + film	ITO on Al_2_O_3_ ceramic	50	45 × 30	ITO	Capacitance	200–1200 °C	170 kHz/°C	Loop coil	Pt	High temperature resistance
[135] 6e	Harsh environment	Strain	Single: Square spiral line + film	–	38	21 × 19	MWCNT/epoxy composite	Frequency	0–71%	gauge factor: 14.7	Patch spiral coil	–	Simple structure
[136] 6f	Harsh environment	Crack	Single: Square spiral line/IDE	Cr/Au on PI	100	10 × 10	Cr/Au	Resistance	0–4%	9.5 MHz/%	Loop coil	Composite	Cost-effective and lossless
[137] 7a	Harsh environment	Temperature Pressure Humidity	Single: Square spiral line/IDE + film/microstructure	Cu on ceramic	T: 40 P: 33 H: 70	42 × 42	T: dielectric material P: Cavity H: PI film	Capacitance, Resistance	T: 25–200 °C, P: 70–220 kPa, H: 24%–90%RH	T: 9.143 kHz/°C P: 3.25 kHz/kPa H: 20 kHz/%RH	Loop coil	–	Monitor multiple parameters simultaneously
[138] 7b	Harsh environment	Temperature Pressure	Single: Square spiral line/IDE + film/microstructure	Ag on ceramic	T: 58 P: 168.2	19.5 × 19.5	T: dielectric material P: Cavity	Capacitance, Resistance	T: 50–500 °C, P: 140–850 kPa	T: 0.062% dB/°C P: 1.16 kHz/kPa	Loop coil	–	Monitor multiple parameters simultaneously

### Biomedical Monitoring

Passive wireless systems stand out in biomedical applications owing to their battery-free operation and minimal electromagnetic radiation, making them highly suitable for complex monitoring scenarios. These systems are divided into implantable and wearable devices, each offering distinct advantages. Implantable applications enable the wireless in vivo monitoring of various physiological parameters without stimulating the human body, while wearable devices allow for continuous, noninvasive monitoring, maintaining real-time tracking of health metrics [27]. These characteristics underline the versatility and safety of passive systems for medical applications.

#### Implantable Devices

Implantable wireless sensing devices, designed for placement within the human body, offer significant advantages over traditional wired monitoring systems in healthcare, which often encounter functional limitations and may cause bodily harm owing to their wires. Passive wireless systems eliminate the need for wired connections or device retrieval, do not require power supplies, and minimize interference with biological processes. These systems meet strict biocompatibility and implantation standards to ensure signal penetration through biological tissues and reliable wireless external readouts after attenuation [20]. Current research is directed toward stable in-body physiological signal detection, real-time monitoring of specific bodily fluid components, and feedback mechanisms for bodily state regulation upon detecting abnormal signals.

##### Physiological Parameters

Given the complex structure and composition of the human body, the precise monitoring of specific physiological parameters crucial for health assessments—such as intracranial pressure, heart relaxation, blood vessel blockage, and bladder pressure—is necessary [15, 19, 33]. These parameters require implantable devices at specific locations for accurate monitoring. This section delves into the design and implementation of implantable passive wireless systems tailored for different regions of the body, detailing their construction and application and emphasizing their role in providing accurate and location-specific health data. Table 2 summarizes various applications of LC passive wireless systems for internal implantation, including the monitoring of physiological parameters and body fluid composition. These systems must rigorously account for the biocompatibility of both the functional sensing structures and the sensing films.

**Table 2:** Passive wireless sensing systems for implantable biomedical monitoring

References	Application		Sensor module		Working frequency (MHz)	Dimension (mm)	Sensitive structure/material	Response type	Sensing range	Sensitivity	Readout module		Characteristic
	Scenario	Target	Structure	Material							Structure	Material	
[139] 8a	Physiology	Intracranial pressure	Cascade: folded square spiral + structure	Mg on PLGA	554	6.4 × 6.4	Structure: air cavity	Capacitance	0–40 mmHg	1 MHz \ mmHg	Loop coil	–	Minimal hysteresis, fast response times, excellent stability, and robustness
[45] 8b	Physiology	Hemodynamics: pressure, pulse rate and flow	Stent Au on stainless steel pipe	Au on PI	100	5 × 0.3	AgNP/PDMS hemispherical microstructure	Capacitance	Pressure: 0–130 mmHg, pulse rate: 0–120, flow: 250–650 ml/min	–	Loop coil	–	Soft, stretchable sensor
[141] 8c	Physiology	Arterial pressure	SU-8 Stent embedded square spiral	Ti/Cu on SU-8	105	3 × 6	Structure: air cavity	Inductance, Capacitance	0–100 mmHg	25 kHz/mmHg	Planar: spiral coil	–	Excellent structure, stable sensitivity
[142] 9a	Physiology	Arterial pressure, blockage	Single: Square spiral/IDE + structure	Cu on PI	350	55 × 6	Structure: PDMS pyramidal	Capacitance	Blockage:0–100% Pressure:0–12.52 kPa	Blockage: 0.05 MHz/% Pressure: 3 MHz/kPa	Planar: patch antenna	–	Flexible configuration, fast response and real-time
[144] 9b	Physiology	Pressure	Single: Circle spiral + structure	Mg on PI	310	5	Structure: PLGA cavity	Capacitance	0–256 mmHg	200 kHz \ mmHg	Loop coil	–	Quantitative, high-resolution
[146] 9d	Physiology	Bladder pressure	Single: U-shape embedded Cu pattern + MEMS capacitor	Cu on 8-Fr stent	65	100 × 0.9	MEMS capacitor	Capacitance	0–120 mmHg	1.3–3.5 kHz/mmHg	Antenna with integrated circuit	–	Constant, low-cost pressure sensing
[70] 10a	Body fluids	Glucose	Single: Square spiral/IDE + film	Ag on PCLAU	800	6 × 6	Film: PAPBA/GOx/GO	Capacitance Resistance	0.5–14 mM	0.35 dB /mM	Loop coil	–	Highly sensitive, specific and reversible sensing properties
[147] 10b	Body fluids	Glucose	Cascade: Folded SRR + hydrogel sandwich	Metal conductive sheet	550	5 × 5	Hydrogel: PBA	Capacitance	10–400 mg/dL	304 kHz/(mg/dL)	Loop coil	–	Ultra-small size, high response
[148] 11a	Implantable: Feedback	Brain tumor	Single: Circle spiral + film	Mg on PI	–	12 × 12	PLA/PI, OST/DOX	–	–	–	Coil: solenoid	–	Flexible, sticky, biodegradable

**Fig. 8:**
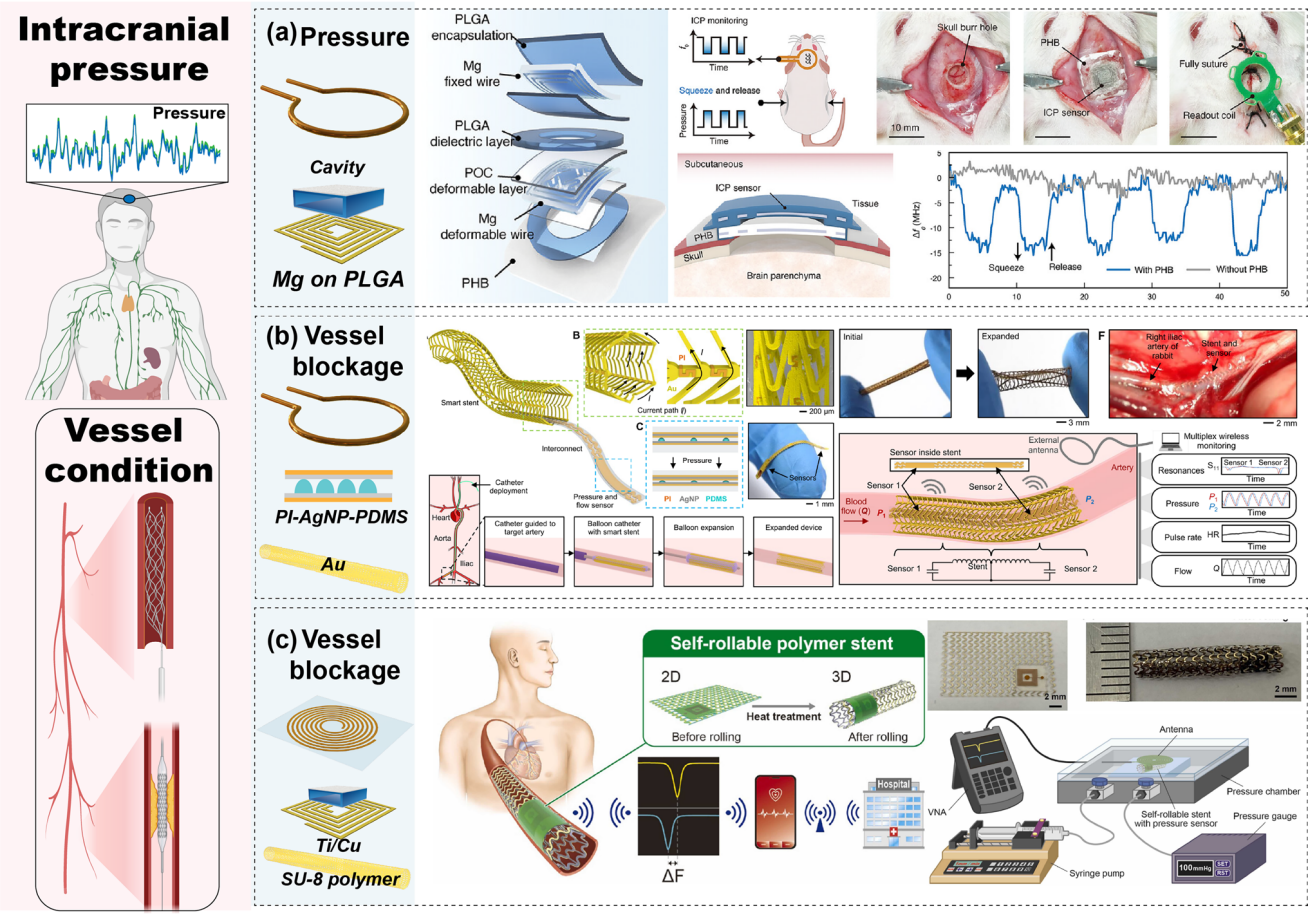
Implantable devices for physiological signal detection. **a** Brain ICP real-time monitoring. Reproduced with permission from Ref. [139]. Copyright © 2024 John Wiley and Sons. **b** Cardiac vascular blockage signal monitoring. Reproduced with permission from Ref. [45]. Copyright © 2022 The Authors-Published by American Association for the Advancement of Sciences. **c** Self-rolling cardiac vascular signal monitoring stent. Reproduced with permission from Ref [141]. Copyright © 2022 Elsevier

Continuous monitoring of intracranial pressure (ICP) is crucial for the diagnosis of life-threatening conditions caused by elevated intracranial pressure. Lin et al. have developed a wireless, biodegradable ICP sensor with a multilayer structure (Fig. 8a) [139]. The sensor comprises an antenna designed with a magnesium (Mg) helical coil on a poly(lactic-co-glycolic acid) (PLGA) substrate and a pressure-sensitive air cavity made of Mg-PLGA-poly(octanediol citrate) (POC). This air cavity responds to external pressure changes by reducing the cavity distance and significantly increasing the capacitance, which translates into a frequency shift detectable by an external wireless coil. The system reliably captures and monitors ICP signals, ensuring timely intervention for critical conditions.

**Fig. 9:**
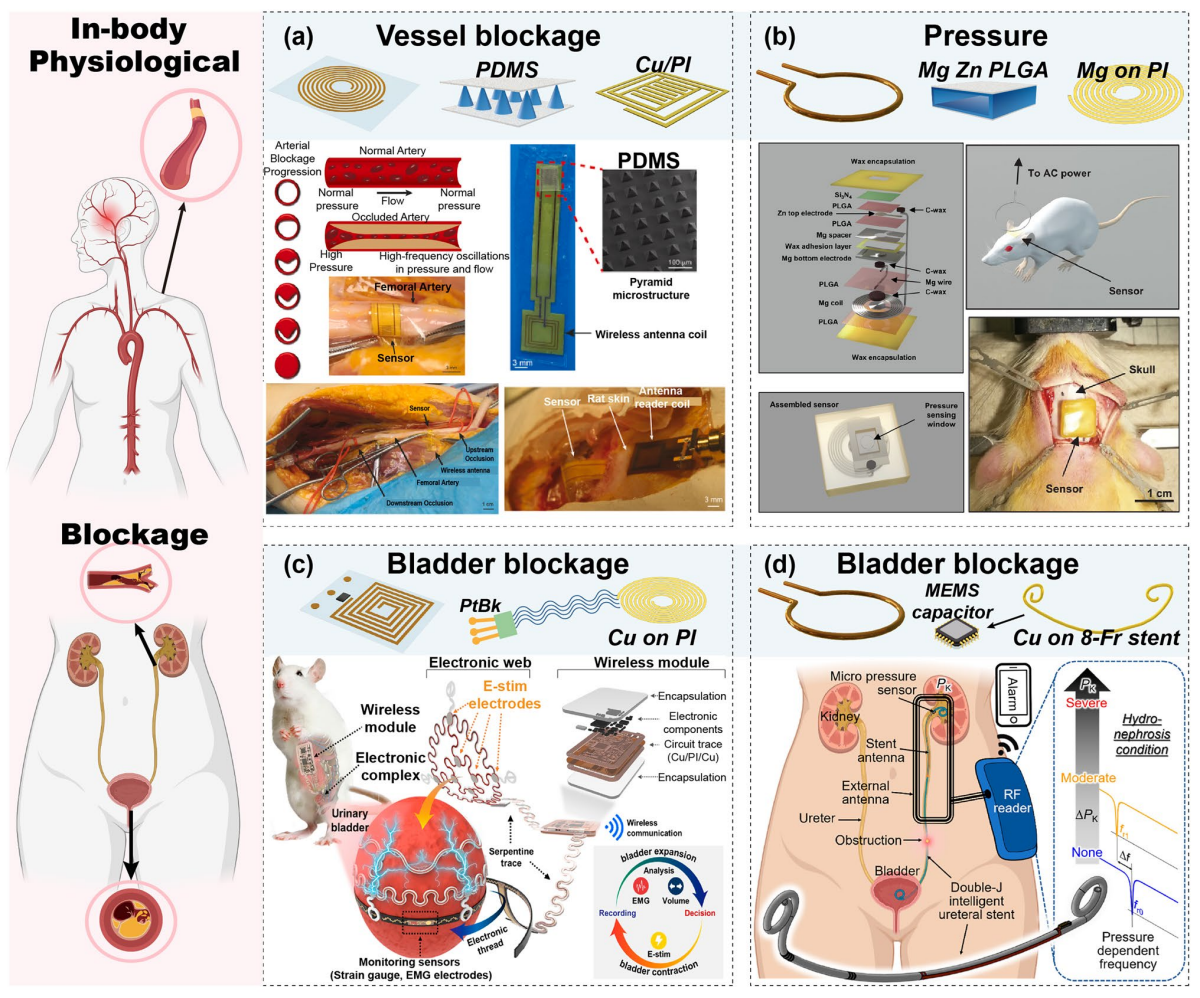
Implantable devices for physiological signal detection. **a** Wrap-around vascular monitoring system. Reproduced with permission from Ref. [142]. Copyright © 2021 Elsevier. **b** In vivo pressure signal monitoring system. Reproduced with permission from Ref. [144]. Copyright © 2020 John Wiley & Sons Inc. **c** Bladder pressure stent-based monitoring system. Reproduced with permission from Ref. [145]. Copyright © 2023 American Chemical Society. **d** Bladder blockage monitoring system. Reproduced with permission from Ref. [146]. Copyright © 2023 American Chemical Society

The heart, one of the most critical organs in the human body, requires monitoring for blood vessel blockages to prevent the obstruction of oxygenated blood delivery [140]. This is particularly crucial for patients with coronary artery disease, where stents are implanted to open narrowed arteries. Robert et al. reported an implantable wireless vascular electronic system comprising a conductive Au ring multi-material inductive scaffold and a nonconductive PI-printed soft-sensor (Fig. 8b) [45]. The passive monitoring stent responds to and monitors arterial pressure, pulse rate, and flow in real-time originating from its silver nanoparticles/polydimethylsiloxane (AgNPs/PDMS) hemispherical microstructure, exhibiting changes in stent inductance and sensor capacitance. This generates resonance frequency offsets that a single-ring readout coil wirelessly reads. For more flexibility, Oyunbaatar et al. investigated a self-coiling polymer vascular stent monitoring system (Fig. 8c) [141]. The passive serpentine stent, made primarily of chromium/gold (Cr/Au) on SU-8, self-coils into a stent-like shape under temperature excitation, causing inductive–capacitive changes through cavity in response to different media and generating a resonance frequency drift. A specific microstrip antenna wirelessly reads the resonance frequency change to determine the vascular status. In addition to implantable stents used for monitoring vascular status, devices wrapped around the outside of the vessel are another common form of monitoring arterial dimensions and occlusions. Ruth et al. developed a wireless capacitive sensor wrapped around an artery during surgery for continuous postoperative arterial health monitoring (Fig. 9a) [142]. Their passive sensor, created by depositing a copper (Cu) pattern on a PI substrate, consists of a spiral wire in cascade form and a fork-finger capacitor with a PDMS pyramidal microstructure encapsulated as a sensitive layer on the IDE. Wrapping the sensor around a blood vessel varies the capacitance in response to changes in external pressure, indicating the degree of vessel blockage and generating a frequency offset read wirelessly by a microstrip antenna. Moreover, measuring pressures in closed cavities and lumens of the body, such as the intracranial space, abdominal cavity, arteries, and osteofascial cavities, can provide crucial diagnostic information for many life-threatening conditions [143]. Lu et al. developed a multilayered bioabsorbable implantable wireless pressure sensor with Mg-based helixes incorporating PLGA pressure-sensing membranes as sensing components, encapsulated by wax (Fig. 9b) [144]. The system responds to external pressure, manifesting as a change in capacitance, which in turn generates a change in the resonance frequency, read wirelessly by a readout coil.

Ureteral obstruction is commonly detected after ureteral stent implantation. It detects pressure in the vesicoureteral canal and can effectively prevent complications, such as hydronephrosis. Lee et al. developed an electrical neuromodulation Cu/PI stent system for urinary bladder monitoring (Fig. 9c) [145]. The platinum black (PtBk) probe sensor, implemented using strain gauges and electromyography electrodes, monitors various physiological parameters, which are then converted into electrical signals and read by the wireless module. The incorporation of a feedback system enables direct electrical stimulation for control. Mohammad et al. proposed an electromechanically functional ureteral stent for ureteral obstruction monitoring (Fig. 9d) [146]. This passive 8-Fr stent consists of an inductive loop (double-J shape) Cu antenna and a MEMS capacitive pressure sensor that matches the shape of the ureter's tubing and produces a change in capacitance in response to changes in bladder pressure. This response, in turn, produces a resonance frequency shift and is read wirelessly by the coil, allowing accurate real-time characterization of ureteral blockage.

##### Body Fluids

**Fig. 10:**
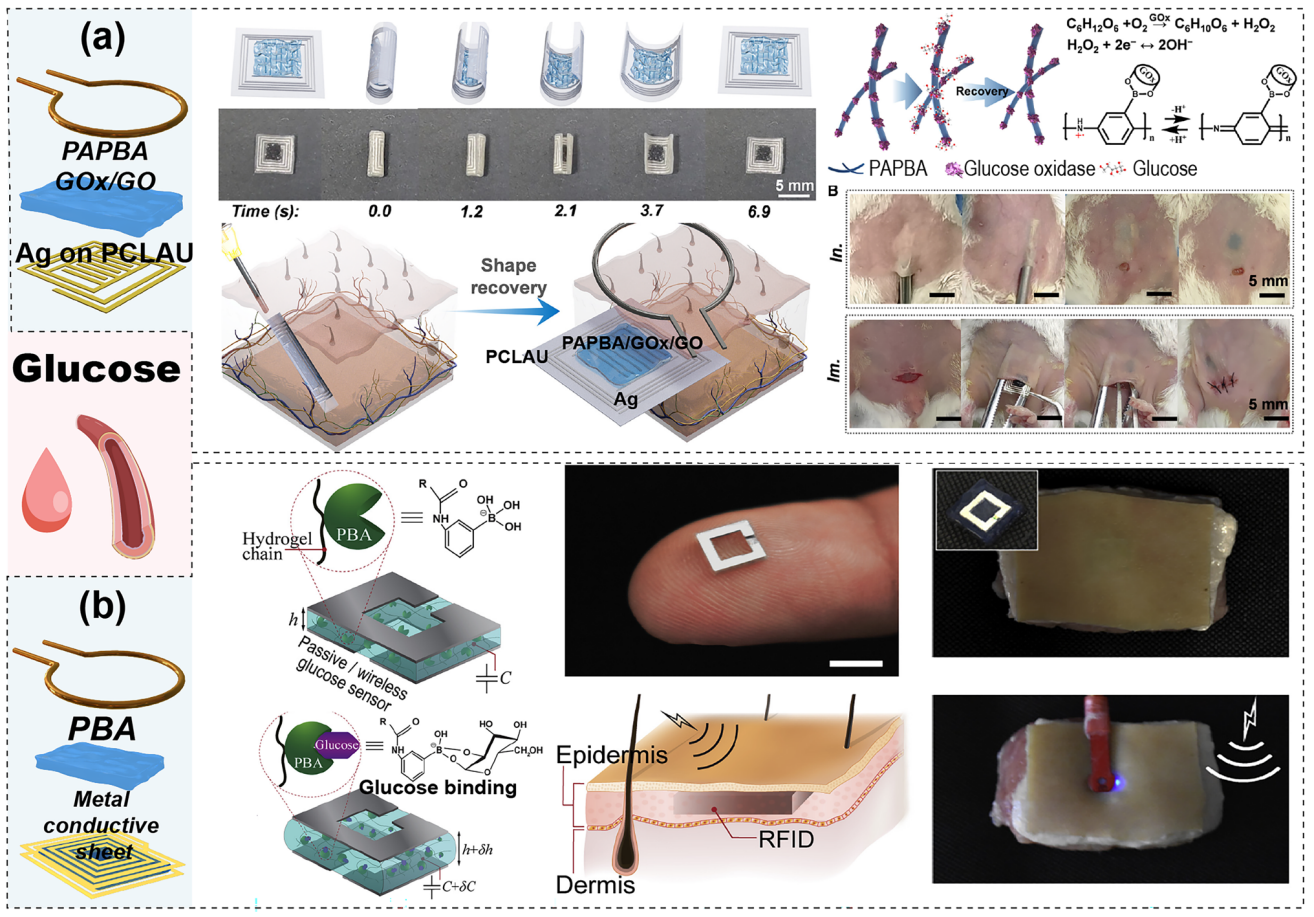
Implantable devices for body fluid components detection. **a** SMED glucose monitoring system. Reproduced with permission from Ref. [70]. Copyright © 2023 Elsevier. **b** PBA hydrogel sandwich folded structure for glucose detection. Reproduced with permission from Ref. [147]. Copyright © 2020 Elsevier

Implantable devices for body fluid monitoring play a crucial role in healthcare, enabling the continuous monitoring of a range of body fluids, tracking metabolic markers and electrolyte levels, and providing essential insights into various physiological and biochemical states. Jiang et al. developed an implantable wireless blood glucose monitor made of shape memory electronics (SMED) that can be folded, minimally invasively implanted, and heated to unfold and function **(**Fig. 10a) [70]. The passive device was fabricated by printing LC spirals embedded with Ag interdigital capacitors as patterns on a shape memory poly(D,L-lactide-co-caprolactone)-based (PCLAU) layer coated with a poly(3-aminophenylboronic acid) (PAPBA)/glucose oxidase (GOx)/GO sensing layer. The sensing membrane responds to glucose and exhibits a change in resistance as its level changes, resulting in resonance frequency variations. Using a wireless readout coil, glucose levels in the body can be measured externally. Manik et al. developed an implantable radio-frequency (RF) resonator based on a phenylboronic acid (PBA) hydrogel sandwich structure for implantable glucose sensing **(**Fig. 10b) [147]. The sensor was a SRR cut from a metal conductive sheet, with the interlayer being a PBA glucose hydrogel. The entire structure can be modeled as capacitive and inductive circuits. The thickness of the hydrogel responds to the glucose concentration, causing changes in the circuit capacitance and shifts in the resonance frequency, which is then read out by wireless readout coils.

##### Feedback Therapy

Implantable devices can integrate therapeutic modules beyond mere sensing, offering a synergistic closed-loop approach. These devices continuously monitor physiological parameters or disease markers and, based on these data, execute treatment interventions such as targeted drug delivery or electrical stimulation. This integration enables sensitive and personalized medical care, aiming to improve patient outcomes through timely and specific therapeutic responses.

**Fig. 11:**
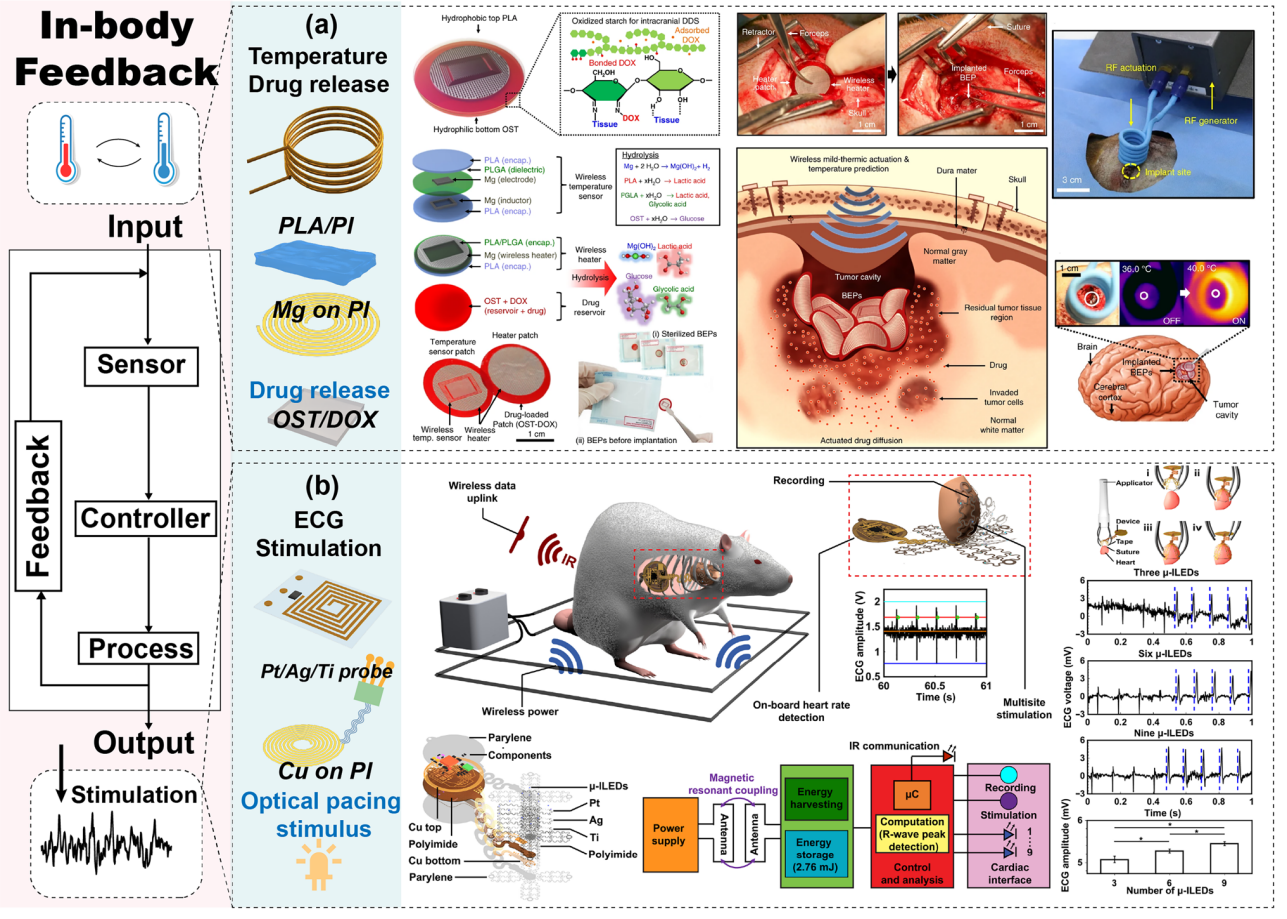
Implantable device integrating sensing and therapeutic feedback. **a** Brain tumor drug delivery device. Reproduced with permission from Ref. [148]. Copyright © 2019 Springer Nature. **b** Passive radio stimulation device. Reproduced with permission from Ref. [149]. Copyright © 2022 The Authors-Published by American Association for the Advancement of Sciences

Implantable devices with basic sensing capabilities can incorporate therapeutic functionalities to establish collaborative closed-loop systems. They adeptly monitor physiological parameters or disease markers, leading to precise therapeutic interventions tailored to individual medical profiles, such as drug release or electrical stimulation. This advanced approach enhances patient care by ensuring responsive and personalized treatment. Lee et al. developed a wireless Mg/PI device for brain tumor drug delivery that could flexibly and conformally adhere to the surgical site of the brain **(**Fig. 11a) [148]. The sensor has a multilayer structure with a magnesium helical coil pattern, forming a wireless heater for mild thermal drug delivery actuation, a temperature sensor for controlled mild thermal actuation, and a flexible drug-carrying patch formed by oxidized starch (OST) containing doxorubicin (DOX). The multi-turn readout coil can be wirelessly excited to heat the heater, inducing drug release at a mild temperature of 42 °C. In addition to drug release, electrical stimulation of the heart is crucial for saving lives. Therefore, Jokubas et al. developed a passive radio stimulation device for the heart with onboard computation for real-time cardiac control by multisite stimulation (Fig. 11b) [149]. The passive device consists of microcircuit modules, helix Cu patterns, and serpentine interconnections connecting the thin-film matrix layers. Platinum (Pt), Ag, Titanium (Ti), and PI are the primary materials that can be integrated into the heart, allowing the monitoring of the activity state of the heart under magnetic resonance-coupled excitation transmission of RF power from an external wireless antenna. The data are analyzed, and electrical stimulation is generated by the microcircuit module for continuous control of cardiac function.

#### Wearable Devices

Compared to implantable devices, wearable sensors offer a noninvasive approach to monitoring various physiological parameters, enhancing user acceptability [34]. Passive wireless sensing systems eliminate bulky power sources, rigid components, and wired connections, thereby improving wearability. Designed to be biocompatible, highly conformal, lightweight, and compact, these sensors provide rich and diverse surface-level human body data. They encompass physiological signals, biomechanical states, motion patterns, and surface fluid component levels, enabling comprehensive real-time health monitoring across multiple scenarios. Table 3 summarizes various applications of LC passive wireless systems in wearable devices, including monitoring physiological parameters and surface body fluids. These systems feature a diverse range of sensing structures, typically based on flexible substrates. They can be integrated with other techniques, enabling a wide array of extended applications.

**Table 3:** Passive wireless sensing systems for wearable biomedical sensing applications

References	Application		Sensor module		Working frequency (MHz)	Dimension (mm)	Sensitive structure/material	Response type	Sensing range	Sensitivity	Readout module		Characteristic
	Scenario	Target	Structure	Material							Structure	Material	
[150] 12c	Wearable: Physiology	Eye pressure	Single: Circle spiral line + structure	Cu on PET	99	–	Structure: Ecoflex pyramid	Capacitance	–	1.101‰ /mmHg	Loop coil	–	Portable and highly sensitive
[151] 12d	Wearable: Physiology	Eye pressure	Single: Circle spiral line + structure	AgSEBS on commercial lens	240	13	Structure: AgSEBS + Silbione + AgSEBS + PDMS	Capacitance	10–40 mmHg	0.030 MHz /mmHg	Coil: solenoid	–	Excellent measurement accuracy, repeatability and user comfort
[152] 12e	Wearable: Physiology	Eye pressure	Single: Circle spiral line + film	Cu	68	13.8	Film: Bubble-filled PDMS	Capacitance	0–39 mmHg	1.15 ‰/mmHg	Loop antenna	–	Simple design and reliable components
[154] 13a	Wearable: Physiology	SCG, pulse and heart rate	Cascade: Circle spiral + electrode	Cu on Ecoflex + PI	24.5	25*50	Cu electrode	Capacitance	0–0.78%	2 MHz/% ε	Planar: spiral coil	–	No batteries required
[157] 13c	Wearable: Physiology	Pulse	Cascade: Folded spiral + hydrogel sandwich	Ag ink on PET	90	–	Hydrogel: IAH microspheres	Capacitance	25–1000 Pa	9.32% /kPa	Loop coil		Excellent perceptual performance
[158] 13d	Wearable: Physiology	Pulse	Cascade: Folded spiral + hydrogel sandwich	Cu on PI	450	–	Hydrogel: Ionic LiCl/HEA/EOEOEA	Capacitance	0–65 kPa	95.2 kHz/kPa	Loop coil	Cu	High sensitivity, LOD, fast response time
[160] 13f	Wearable: Physiology	Humidity	Single: Circle spiral line/IDE + film	Al on parchment	50	–	Al_2_O_3_	Capacitance	0–85% RH	87 kHz RH^−1^	Loop coil	Cu	Good repeatability and stability
[161] 14a	Wearable: Biomechanical	Stress pressure	Cascade: Square spiral line + film	Al on PI	75	–	Film: Au/AgNW/PI MXene-PDA PDMS	Capacitance	0–30 kPa	2 MHz/% strain	Planar: spiral coil	Al/PI	High linearity and adjustable sensitivity
[84] 14b	Wearable: Biomechanical	Finger tactile	Cascade: Folded vertical spiral line + structure	Al or Au on PI	100, 200, 300	–	Structure: PPy-coated PDMS pyramids	Capacitance	0–10 kPa	0.32–0.039 dB /kPa	Loop coil	–	Acquire and process multiple sensor signals in parallel
[87]14c	Wearable: Biomechanical	Pressure	Cascade: Folded spiral line + structure sandwich	Cu on Ecoflex	280	–	Structure: MWCNT/PDMS pyramids	Capacitance	0–1000 Pa	8 MHz/kPa	Planar: spiral coil	–	Flexibility and fast tactile recognition
[83] 14d	Wearable: Biomechanical	Insole pressure	Cascade: Square spiral line + film	Ag on flexible composite protein film (FCPF)	620	8*8	Film: FCPF-Ag-Ecoflex-Ag-FCPF	Capacitance	0–419 kPa	1.456 MHz/kPa	Loop coil	–	Good cycle stability and high sensitivity, compact
[162] 14e	Wearable: Biomechanical	Insole pressure	Single: Circle spiral line/IDE + structure	Ag on PI	2700	17	Structure: Miura-ori origami structure	Frequency	0–40 kPa	2.1 MHz/kPa	Planar: patch antenna	–	Cost-effective, personalized health monitoring
[163] 15a	Wearable: Motion	Human motion	Cascade: Folded 3D spiral line + film sandwich	LM	30	60*60	Film: Ecoflex	Resistance	0–160% strain	1.3%/kPa	Loop coil	–	Low detection limit, high quality factor
[164] 15b	Wearable: Motion	Human motion	Cascade: Folded spiral line + film sandwich	Au on PDMS	160	9*9	Gr/PDMS film	Capacitance	0–100 kPa	0.0078–0.24/kPa	Loop coil	–	Excellent stability and durability
[165] 15c	Wearable: Motion	Human motion	Cascade: Folded spiral line + film sandwich	Cu on PI	13.56	–	Film: Fabric ferrite	Capacitance	0–15 kPa	1.23 × 10^−2^ /kPa	Antenna with integrated circuit	–	Fast, real-time data analysis and processing capabilities
[166] 15d	Wearable: Motion	Human motion	Cascade: Folded spiral line + structure sandwich	Cu on PI	45	10*10	Structure: Ferrite film 3D microstructured fabric gasket	Capacitance	0–20 kPa	0.19 MHz /kPa	Planar: spiral coil	–	Excellent reproducibility
[57] 15e	Wearable: Motion	Human motion-strain	Single: Circle spiral line + film	Conductive thread on polyester-spandex	13.56	31	Film: Ecoflex + carbon black + PET	Resistance	0–100%	–	Antenna with integrated circuit	–	User comfort
[167] 15f	Wearable: Motion	Human motion-strain	Single: Square spiral line	Ag ink on PDMS	14	95*20	Ag electrode	Frequency	0–120%	86 kHz/%	Planar: spiral coil	–	High compatibility
[168] 15 g	Wearable: Motion	Human motion-strain	Cascade: Unfolded spiral line + film	Liberator thread on spandex fabric	15	80*60	Film: Conductive spandex-Non-conductive spandex-Conductive spandex	Capacitance	5%–70%	30 kHz/%	Loop coil	–	Fast sampling rate
[169] 15 h	Wearable: Motion	Human motion-strain	Single: Spiderweb shape line	LM on silicone substrate	13.54	26	LM	Inductance	0–300%	5.7 kHz/%	Coil: solenoid	Cu	Highly stretchable and stable in deformation
[170] 16a	Wearable: Body fluid	Eye tears- glucose	Cascade: Circle spiral + optoelectronic module	AgNFs on Elastofilcon	50	12	Module: LED + rectifier + diode + GOD	Resistance	0–0.9 mM	22.72%/mM	Loop coil	–	Real-time wireless
[171] 16b	Wearable: Body fluid	Eye tears- glucose	Cascade: Circle spiral + FET module	Graphene-AgNW hybrid on parylene	4100	14	Module: Graphene-AgNW hybrid S/D	Resistance	1 μM–10 mM	0.5 μA/104 mM	Loop coil	–	Multiplexing, high conductivity, flexibility and transparency
[172] 16c	Wearable: Body fluid	Saliva-pH	Cascade: Folded SRR + hydrogel sandwich	Au	400	2*2	Hydrogel: Porous silk membrane-modified PNIPAM	Capacitance	pH:7–3	50 MHz pH:5 to 3	Loop coil	–	Enhanced sensitivity
[173] 16d	Wearable: Body fluid	Saliva-pH	Cascade: Square spiral + electrochemical probe	Cu on PI	13.56	10*8	Probe: PPy/F on Ag/AgCl electrode	Voltage	pH:3–8	62.97 mV/pH	Antenna with integrated circuit	–	Miniaturized, flexible, and able to conform to the teeth
[174] 16e	Wearable: Body fluid	Sweat-cocaine	Cascade: Circle spiral + electrochemical probe	Cu on PI	13.3	–	Probe: Pd/PdHx	Capacitance	10^–11^–10^–5^ M	1.213 kHz/mM	Coil: solenoid	–	In situ sensing under mild and biocompatible conditions
[175] 16f	Wearable: Body fluid	Sweat-glucose	Cascade: Circle spiral + electrochemical probe	Cu on Ethylene	105	10	Probe: Ag/AgCl (PVB + KCl), Au (ISM)	Voltage	0–600 μM	2.82 MHz/mM	Loop coil	–	Lightweight, compact and portable
[177] 16 g	Wearable: Body fluid	Sweat-pH	Cascade: Square spiral + electrochemical probe	Ag on PI	13.56	23*28	Probe: Ag/AgCl (PANI/carbon)	Voltage	pH:3–10	51.76 mV /pH	Antenna with integrated circuit	–	Accurate, on-demand
[178] 16 h	Wearable: Body fluid	Wound-Staphylococcus aureus	Cascade: Circle spiral + electrochemical probe	Cu on PI	13.56	–	DNA gel	Capacitance	0–10^6^ CFU	0.38 * 10^–3^ mV	Antenna with integrated circuit	–	Lightweight and flexible
[179] 16i	Wearable: Body fluid	Wound-Healing condition	Single stage: Circle spiral line + film	Cu on PI	80	40	Cu electrode	Frequency	0–100%	3.547 MHz/% wound closure	Coil: coplanar loop	–	High reliability
[180] 17a	Wearable: Multiple signals	Strain, humidity, temperature	Cascade: Different patterns	Graphene on PI	13.56	–	Humidity: LIG Temp: LIG Strain: Ecoflex	Resistance	S:0–30% T: 26–50 °C H:90%	T:0.06% /°C H:0.525%/RH	Antenna with integrated circuit	–	Rapid response, remarkable stability
[181] 17b	Wearable: Multiple signals	Pressure, temperature	Cascade: Circle spiral + structure	Cu on PI	13.56	–	P: MPTMS-Rigid pad-Rigid sheet-Tri-layered film cavity, T: Thermistor	Resistance	P:0–10 kPa T:19.7–34°C	P: 2 mV/kPa	Antenna with integrated circuit	–	Long-term stability
[182] 17c	Wearable: Multiple signals	Strain, temperature and moisture	Cascade: Circle spiral + electrochemical probe	Cu on Tegaderm	13.6	74*50	ECG: Cu electrode T: Thermistor H: Cu electrode	Resistance	S:0–30%, T:31–35°C M: 90–30 a.u	–	Loop coil	–	Digital and cost-effective

##### Physiological Parameters

**Fig. 12:**
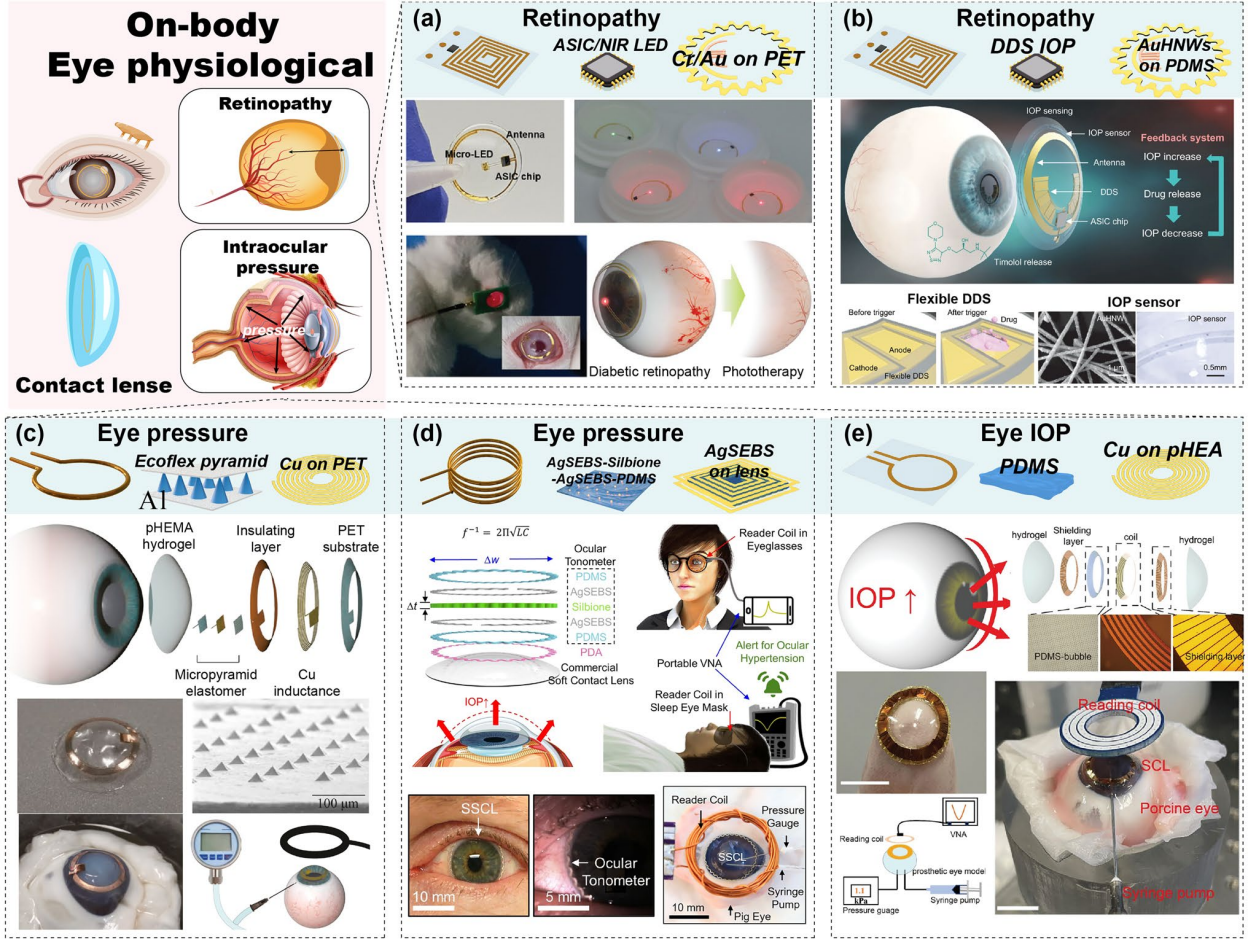
Wearable device for eye physiological information detection. **a** Far-red/NIR luminescent contact lens for retinal treatment. Reproduced with permission from Ref. [150]. Copyright © 2022 John Wiley & Sons Inc. **b** Glaucoma IOP detection-treatment closed-loop contact lens. Reproduced with permission from Ref. [151]. Copyright © 2022 Springer Nature. **c** Hydrogel smart contact lens for wireless IOP monitoring. Reproduced with permission from Ref. [152] Copyright © 2022 American Chemical Society. **d** Commercial contact lens-based IOP monitoring system. Reproduced with permission from Ref. [153]. Copyright © 2022 Springer Nature. **e** Circle-shaped passive wireless IOP contact lens. Reproduced with permission from Ref. [154]. Copyright © 2024 John Wiley & Sons Inc

The surface of the human body harbors a myriad of physiological parameters indicative of health status. Passive wireless systems have made it possible to measure specific physiological signals previously challenging to detect, such as those emanating from the eyes [11, 80]. Wearable sensors, especially those based on contact lenses, present a promising avenue for the continuous and noninvasive monitoring of eye signals. For example, Lee et al. developed a noninvasive, smart, wireless contact lens capable of emitting far-red/NIR light for the repeated treatment of diabetic retinopathy (Fig. 12a) [150]. This contact lens incorporates a light-emitting diode (LED) and a helical resonance sensing module for wireless energy transfer, featuring Cr/Au electrodes on a polyethylene terephthalate (PET) substrate. This system, powered wirelessly by a specially designed circuit, enables LEDs to emit therapeutic red light to the retina, thereby preventing diabetic retinopathy. Advancing this field further, Kim et al. designed an integrated smart contact lens for glaucoma patients, offering both diagnostic and therapeutic capabilities (Fig. 12b) [151]. This lens features a helix-shaped intraocular pressure (IOP) sensor made from sensitive hollow gold nanowires (AuHNWs), a compact flexible drug delivery system, a wireless power and communication system, and a specialized integrated circuit chip for monitoring and controlling IOP in glaucoma. The helix-wire contact lens continuously gauges IOP and administers medication, with IOP-specific data wirelessly transmitted by the designed circuitry. Similarly, Zhu et al. developed a hydrogel-based smart contact lens with enhanced sensitivity for wireless IOP monitoring (Fig. 12c) [152]. The system comprises a Cu helical inductive coil, a pyramidal microstructure, a poly(hydroxyethyl methacrylate) (pHEMA) hydrogel substrate, and a PET substrate with conformal overlap, functioning collectively as an LC circuit. The parallel plate capacitance of the Ecoflex pyramidal microstructure responds to IOP variations by exhibiting changes in capacitance, leading to resonance frequency shifts. A single-loop readout coil can remotely measure this signal, offering potential for clinical IOP monitoring applications. In response to market demand, Zhang et al. undertook the secondary development of a soft contact lens based on a commercial platform without altering its inherent properties (Fig. 12d) [153]. Their innovative smart soft contact lens was designed to conform to various corneal curvatures and thicknesses, enabling precise absolute IOP measurements under dynamic conditions. The passive sensor within this lens consists of Ag serpentine spirals encapsulated in PDMS as a pressure-sensitive material (AgSEBS/Silbione/AgSEBS/PDMS). This structure reacts with the IOP levels, causing capacitance changes and subsequent resonance frequency shifts, which are wirelessly read out via a multiloop coil. Similarly, Yang et al. introduced an IOP wireless sensor embedded in a contact lens, designed for repeated daily use (Fig. 12e) [154]. The sensor comprises a circular Cu spiral coil, two shielding layers, and a bubble-filled PDMS layer, which together form an LCR-type model. The inclusion of bubbles within the PDMS layer enhances sensitivity to IOP changes, causing significant capacitance variations and corresponding frequency shifts, achieving a high sensitivity of 1.15 ‰/mmHg. Consequently, IOP levels can be wirelessly read by the external loop antenna, enabling real-time IOP monitoring with high stability.

**Fig. 13:**
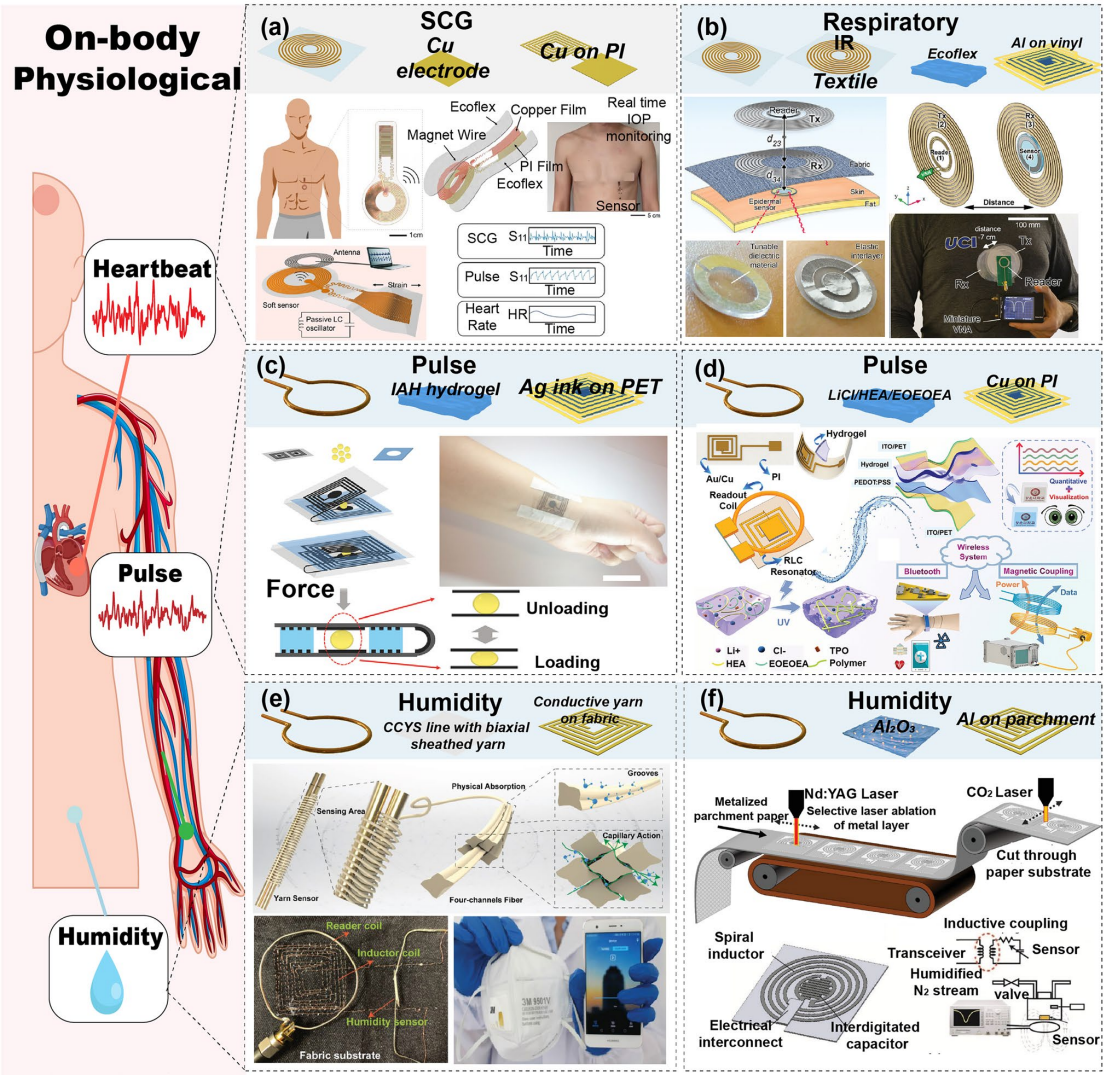
Wearable device for human body physiological information detection. **a** Wireless battery-free soft human heartbeat sensor based on Cu-based PI. Reproduced with permission from Ref. [156] Copyright © 2021 John Wiley & Sons Inc. **b** Wireless heartbeat monitoring system based on enhanced magnetically coupled resonator chain. Reproduced with permission from Ref. [157] Copyright © 2023 The Authors. **c** Wireless pulse monitoring system based on sandwich ionic hydrogel. Reproduced with permission from Ref. [159] Copyright © 2017 John Wiley & Sons Inc. **d** Ionic hydrogel pressure monitoring system. Reproduced with permission from Ref. [160] Copyright © 2023 John Wiley & Sons Inc. **e** Fully textile wireless flexible humidity sensor. Reproduced with permission from Ref. [161] Copyright © 2019 John Wiley & Sons Inc. **f** Wearable passive wireless humidity sensor. Reproduced with permission from Ref. [162] Copyright © 2022 John Wiley & Sons Inc

In addition to monitoring the health status of the eyes, passive wireless wearable devices have been extensively developed to monitor various physiological signals across the human body. These innovative devices can track key physiological parameters such as heartbeat, pulse, and humidity [155]. Monitoring heart rate and pulse is crucial as they offer vital insights into heart health and the overall condition of the circulatory system. Arrhythmia, along with an unusually rapid or slow heartbeat, can indicate potential health issues. Additionally, long-term observation of these parameters is instrumental in managing chronic conditions such as hypertension and heart disease. Consequently, developing physiological signal sensors that can be conformably attached to human skin is an area of significant research interest. Ma et al. developed a wireless, battery-free soft sensor for dynamic cardiovascular health monitoring **(**Fig. 13a) [156]. This passive sensor integrates a capacitive strain transducer with an inductive helical coil made from Cu on a PI substrate, allowing for conformal skin attachment. The LC circuit of the sensor is sensitive to cardiac vibrations in the chest, translating these into capacitance changes, which are then wirelessly transmitted by a microstrip antenna with corresponding dimensions. Furthermore, to enhance the detection of subtle physiological signals, in addition to developing liquid metal (LM) electronic tattoos that adhere to the skin [157], the introduction of IR for signal amplification has been proven to be an effective approach. Amirhossein et al. engineered a magnetically coupled resonator chain for wireless heartbeat monitoring **(**Fig. 13b) [158]. This system employs a secondary structure, where the sensor node is tightly magnetically coupled to the receiver coil and inductively coupled to the transmitter coil. The sensor node, a SRR-based LCR resonator, utilizes Ecoflex 00-30 as an intermediate layer in the pressure sensor. This setup responds to the external stimulation of a heartbeat, manifesting as a capacitive change that induces a resonance frequency shift, which is then wirelessly and accurately captured by a microstrip transmitter coil connected to a VNA via the receiver coil. Compared to heartbeat information, pulse signals are more readily monitored because of their detection location at the wrist and can similarly reflect cardiovascular health. Tai et al. devised a passive wireless pressure device utilizing ionic alginate hydrogel (IAH) microspheres (Fig. 13c) [159]. In this device, the hydrogel is layered between Ag antennas with electrodes on double-sided tape, serving as a bonding frame. This passive device, characterized by a helical shape, functions as an LC circuit. The ionic microspheres act as an interfacial capacitance, altering the response to external pressure and thus changing the resonance frequency. Attached to a bracelet, this sensor allows wireless reading of pulse signals through a single-loop antenna. Similarly, Guo et al. designed a wireless wearable smart patch based on a light-cured ionic hydrogel for real-time passive sensing of human body pressure. The ionic hydrogel layer, stacked in the middle of the electrode as a shared functional layer, exhibits capacitance changes in response to pressure-sensing, producing frequency changes, which a single-loop readout coil can wirelessly read (Fig. 13d) [160]. Humidity monitoring is also of paramount importance. Ma et al. developed an all-textile, wireless, flexible humidity sensor suitable for mask embedding for respiratory monitoring (Fig. 13e) [161]. The sensor comprises functional yarn wound around a Cu wire, creating a spiral wire inductive shape. The functional yarn, which is sensitive to humidity, alters its capacitance, inducing a resonance frequency shift that a single-loop readout coil wirelessly reads. Addressing the need for economic considerations, Gopalakrishnan et al. developed a scalable and rapid fabrication method for cost-effective humidity sensors (Fig. 13f) [162]. Their technique involves the lamination of parchment paper with an aluminum (Al) film, which is subsequently patterned into an interdigital capacitance spiral coil using laser ablation. This coil operates as a passive sensor within the frequency range of several tens of MHz. The laser ablation process generates Al_2_O_3_ nanoparticles at the ablated sites, which act as functional materials with a specific responsiveness to humidity variations. These humidity-induced changes are manifested as shifts in the sensor's resonance frequency, which are wirelessly detected and quantified using a readout coil.

##### Biomechanical Parameters

Biomechanical testing is a comprehensive assessment that evaluates human movement, muscle activity, and force output. It quantifies and analyzes the mechanical behavior of the human body during physical activity. Key components of biomechanical testing include pressure and load analysis, assessing the stresses endured by various body parts under weight-bearing conditions or during movement, and gait analysis, examining foot landing patterns and the distribution of forces.

**Fig. 14:**
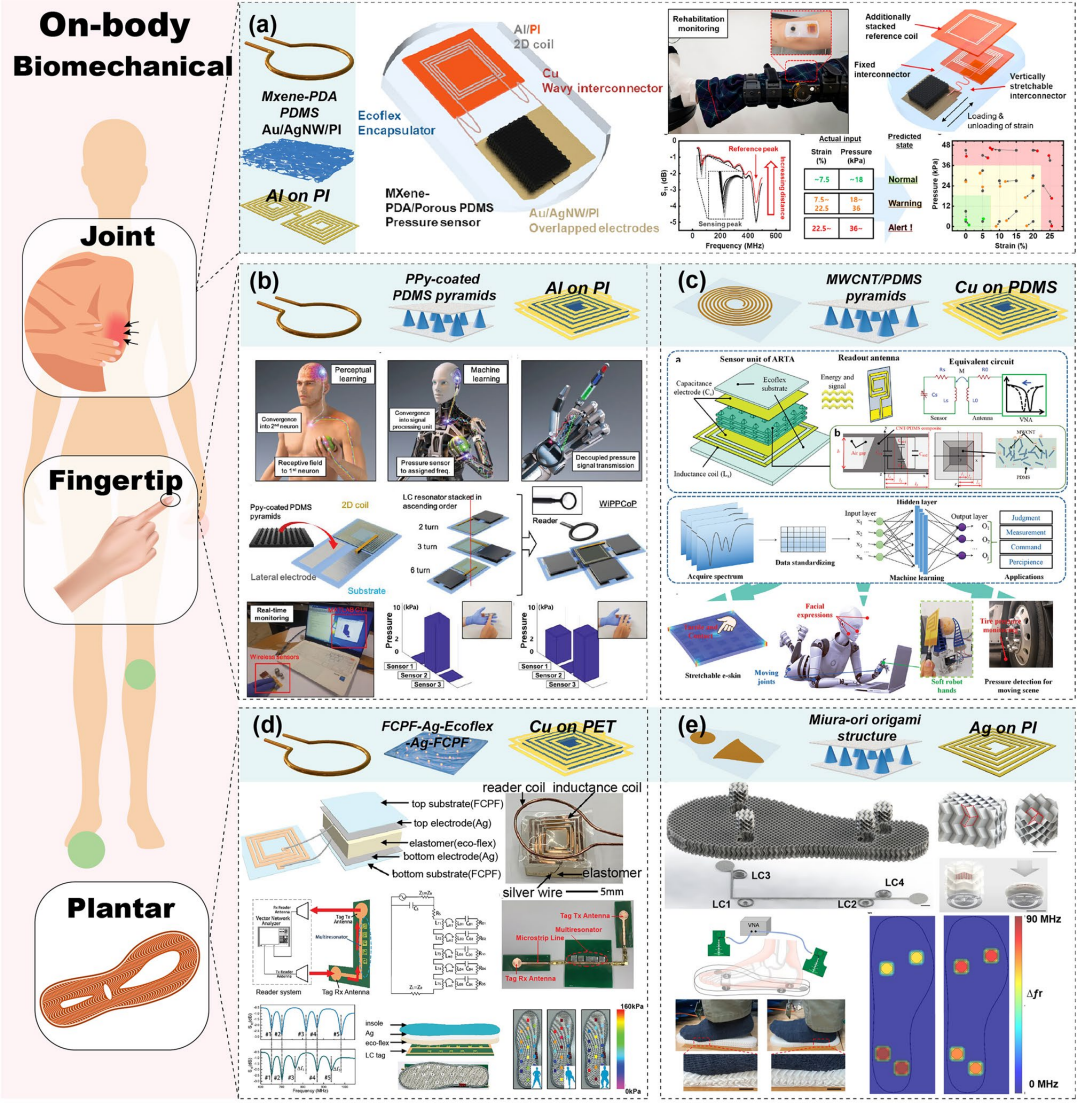
Wearable devices for human biomechanical detection. **a** MXene-integrated wireless stress pressure-sensing platform. Adapted with permission from Ref. [163] Copyright © 2020 American Chemical Society. **b** Wireless finger tactile sensing based on thin polypyrrole layers. Reproduced with permission from Ref. [86] Copyright © 2019 John Wiley & Sons Inc. **c** Wireless array pressure-sensing system incorporating machine learning. Reproduced with permission from Ref. [87] Copyright © 2023 John Wiley & Sons Inc. **d** Array sensing insole based on Ecoflex pyramid microstructures. Reproduced with permission from Ref. [85] Copyright © 2021 John Wiley & Sons Inc. **e** Battery-free wireless Miura-ori origami pressure-sensing insole. Reproduced with permission from Ref. [164] Copyright © 2022 Springer Nature

The biomechanical testing of multiple body parts is significant. For example, Lee et al. developed a MXene-integrated wireless sensing platform capable of differentiating between pressure and strain **(**Fig. 14a) [163]. This differentiation was achieved through the deconvolution of individual signal outputs via a dual-mode wireless signal change mechanism. The sensor device consists of a thin, flexible component with cascaded helices and MXene–polydopamine (PDA)/porous PDMS parallel capacitive plates, modeled as LCR circuits. This device responds to pressure by inducing changes in capacitance and resonance frequency. When mounted on the knee, it can detect and classify the level of mechanical stimuli, with the data read wirelessly by a microstrip antenna, promising for various wireless haptic sensing applications in wearable and implantable electronics.

Research on haptics, especially finger haptics, is crucial for enhancing our understanding of human sensory capabilities. It has significantly improved the precision and naturalness of robotic systems and advances in rehabilitation and prosthetic technologies. Lee et al. developed a wireless multiparallel pressure-sensing platform (Fig. 14b) [86]. This platform features helical wire inductive coils patterned as electrodes with polypyrrole (PPy)-coated pyramidal microstructures of PDMS serving as capacitors. A passive sensor, a stack of multiple helices of varying sizes, responds to pressure by converting it into capacitance changes, leading to variations in resonance frequency. Applied to a finger, this technology can accurately determine applied pressure with further modularization of the sensor matrix, thus enhancing tactile detection. Xu et al. expanded this concept by developing an adjustable tactile sensing matrix resonator with machine learning capabilities **(**Fig. 14c) [87]. Comprising an array of sensing units of different sizes spaced at a frequency of 15 MHz to form an inductor with a pyramidal MWCNT/PDMS microstructure functioning as a capacitor, the matrix can be subdivided into sections for distributed load analysis. Integrating machine learning allows for the identification of tactile positions, applicable in scenarios such as finger touch, with data captured wirelessly using a single microstrip line antenna.

Moreover, gait force analysis provides critical insights into human health and athletic performance by enabling the analysis of foot landing and force application patterns. Wen et al. designed a biocompatible wireless LC pressure-sensing integrated matrix **(**Fig. 14d) [85]. This flexible and compact LC sensor, composed of cascaded silk-based Ag helical inductive wire electrodes and Ecoflex parallel capacitive plates, reflects single pressure points through capacitance changes. These changes are then converted into resonance frequency shifts and wirelessly read using a single-loop readout coil. The integrated matrix of helical units, each with different frequencies and interconnected by microstrip lines, can be shaped like an insole. Capacitance changes in each unit, varying with different frequency shifts due to pressure, reflect the force at various locations. The overall pressure distribution is obtained using a UWB antenna to characterize plantar pressure. To enhance accuracy and prevent errors, Kim et al. developed a 3D-designed, battery-free wireless Miura-ori origami pressure sensor by deploying LC Ag helix inductive sensors at four strategic locations (Fig. 14e) [164]. This sensor, which can be modeled as an LC circuit, undergoes 3D origami morphology and resonance frequency changes in response to pressure variations. The insole resonance frequency, representing the pressure data, was wirelessly read by two UWB antennas, enabling a wireless reading distance of up to 6 cm.

##### Motion Signals

Human motion signals and posture detection are pivotal technologies with significant applications in fields such as health monitoring, rehabilitation medicine, sports science, and human–computer interaction. This type of detection often involves the use of sensors and algorithms to capture, analyze, and interpret signals related to human motion and posture. Signals emanating from various body parts can indicate diverse human conditions and wireless systems facilitate the simplification and deployment of sensors across different body regions. Contemporary research has focused on facial expression detection, finger posture, body networks, and leg posture assessments.

**Fig. 15:**
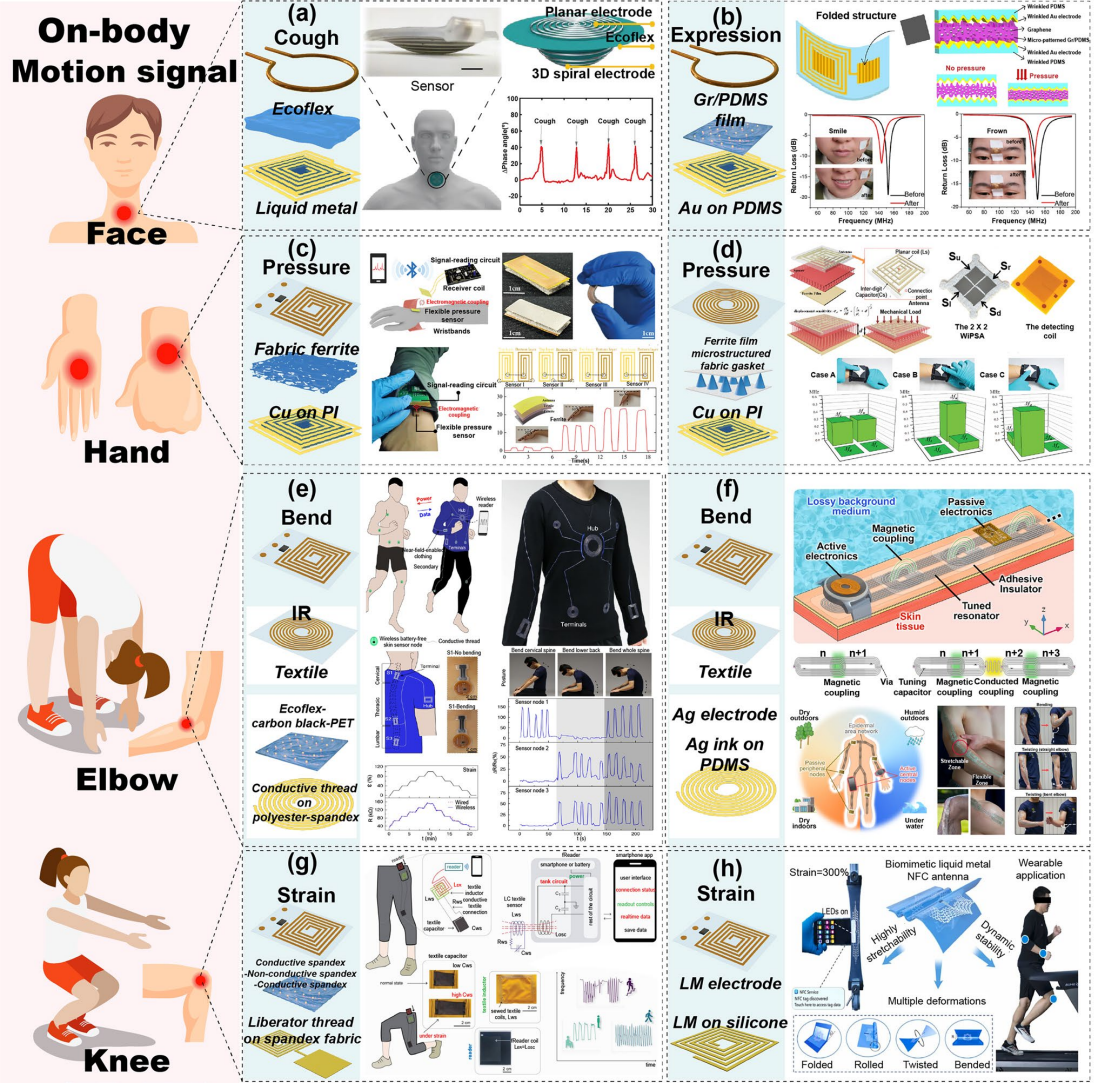
Wearable devices for human motion signal detection. **a** 3D spiral LM wireless sensing system for throat signal detection. Reproduced with permission from Ref. [165] Copyright © 2023 John Wiley & Sons Inc. **b** Micropatterned graphene/PDMS composite wireless flexible system for expression detection. Reproduced with permission from Ref. [166] Copyright © 2018 Elsevier. **c** Fabric-based wireless pressure-sensing system for wrist bending sensing. Reproduced with permission from Ref. [167] Copyright © 2023 John Wiley & Sons Inc. **d** Ferrite film/flexible fabric pressure-sensing system for gesture recognition. Reproduced with permission from Ref. [168] Copyright © 2019 John Wiley & Sons Inc. **e** Battery-free sensor networks based on functional textile pattern interconnections. Reproduced with permission from Ref. [58] Copyright © 2020 Springer Nature. **f** Multi-node wireless networks with textile-integrated metamaterials. Reproduced with permission from Ref. [169] Copyright © 2023 Springer Nature. **g** Smart clothing-based on textile spiral-inductive coils. Reproduced with permission from Ref. [170] Copyright © 2023 John Wiley & Sons Inc. **h** Bionic spiderweb-based LM wireless sensor network. Reproduced with permission from Ref. [171] Copyright © 2023 Elsevier

Starting with facial expression detection, Qu et al. developed a wearable 3D helical LM sensing system **(**Fig. 15a) [165]. Characterized by a rotating top-shaped structure, this system consists of an LM planar helical inductive wire embedded in an Ecoflex elastomer, complemented by a 3D helix. When subjected to external loads, the passive sensing system experiences deformation in the spiral gyro-shaped structure, leading to capacitive–inductive changes and consequent resonance frequency shifts, which are then wirelessly read using a single-loop readout coil. This system is adept at recognizing cough signals when attached to the throat. Similarly, Kou et al. developed a flexible wireless pressure sensor using micropatterned graphene/PDMS composites **(**Fig. 15b) [166]. Situated within a folded PDMS substrate and a continuous Au helix pattern, this composite forms an LC circuit. When applied to the cheek, a thin-layer sensor can detect smiling behavior, and when applied to the middle of the forehead, it can detect frowning movements. All these signals are wirelessly read out via a single-loop readout coil, providing fast and sensitive detection.

Joint flexion due to movements such as wrist rotation, finger gestures, or touch is also a crucial aspect of motion detection. Yan et al. developed a wireless pressure-sensing Cu PI-based system based on LC resonators for real-time flexible contact pressure measurements (Fig. 15c) [167]. The passive sensor comprises a sandwich structure with a double-layer planar helical induction coil, a deformable fabric layer, and a ferrite film. Affixed to the wrist, this sensor responds to the degree of wrist flexion and is read using a wireless circuit. Conversely, Nie et al. developed a ferrite film/fabric spacer pressure-sensing system based on a stacked Cu PI-based structure (Fig. 15d) [168]. The thin-layered flexible sensor, when made into a 2 × 2 matrix and attached to multiple body locations, particularly the wrist, can detect force and posture at four wrist positions. These postural signals are wirelessly read by a readout antenna to monitor human health and aid in early disease diagnosis.

Furthermore, constructing body sensor networks to detect comprehensive information about multiple actions and human movement patterns can offer personalized monitoring and analysis beyond the capabilities of individual sensing nodes. Recent advancements in integrating sensors into textile garments have led to the rapid development of body sensor networks. Lin et al. designed a passive system based on battery-free sensor networks interconnected with functional textile patterns integrated into garments (Fig. 15e) [58]. This system, deploying inductive coils at the sensing nodes and utilizing a textile-on-garment network as a signal repeater, is read wirelessly using a readout circuit. It is flexible, adaptable, and capable of monitoring cervical and spinal curvatures. Hajiaghajani et al. created a biocompatible, magnetically sensitive metamaterial chain on garments (Fig. 15f) [169]. This network, which can be placed directly on the skin and operates seamlessly, is a body-wide network composed of biocompatible stretchable conductive Ag ink. Passive sensors are configured at joint bends to detect postures, including bending and twisting, with data transmitted through a serpentine-shaped human body network and read wirelessly. These body-region networks are secure, customizable, and have immediate scalability potential. The deployment of textile smart clothing-based electronics is flexible and can be configured on demand for each application scenario. Galli et al. developed a wireless sensing wearable smart clothing integrated into trousers tailored to human movement scenarios (Fig. 15g) [170]. This system, comprising cascaded fabric helical inductive coils and parallel textile capacitors, responds to different body movements and generates a frequency offset, which is wirelessly read by either a single-loop readout coil or a circuit that determines movement posture. He et al. developed a stretchable, deformation-stable, wirelessly powered antenna based on a bionic spiderweb structure and an ultrathin LM microchannel (Fig. 15h) [171]. The sensor's spiderweb morphology, modeled as an LC circuit, varies its inductance in response to external stress, leading to changes in resonance frequency. These sensors, conformally fit to the hand, wrist, and knee joints, monitor deformation in these areas and determine body posture, which is then wirelessly read by the readout circuit.

##### Body Fluids

Wearable devices that detect epidermal body fluids represent an emerging technology with significant potential for health monitoring, disease diagnosis, and exercise science [10, 14]. These devices provide critical insights into an individual’s health status by analyzing biomarkers on the skin surface or within body fluids. Presently, commonly used tests for body surface fluids include the analysis of tears, saliva, sweat, and wound exudates.

**Fig. 16:**
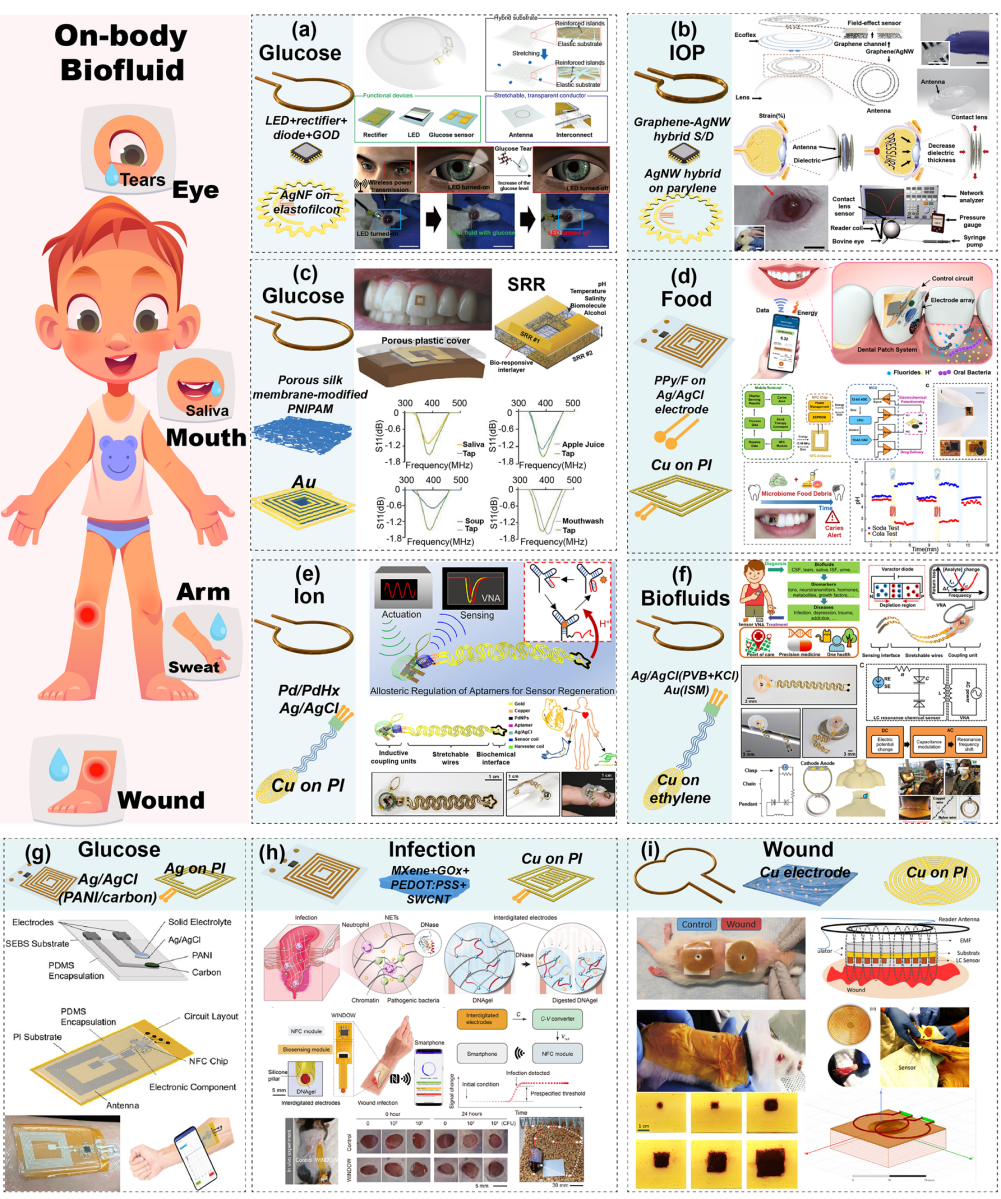
Wearable devices for biofluid detection. **a** Soft smart contact lens-based on nanostructured helical inductive wires for glucose monitoring. Reproduced with permission from Ref. [172] Copyright © 2017 The Authors-Published by American Association for the Advancement of Sciences. **b** Wearable contact lenses based on graphene and AgNWs helical wire inductors for simultaneous glucose and intraocular pressure (IOP) monitoring. Reproduced with permission from Ref. [173] Copyright © 2017 Springer Nature. **c** Silk film/PANIPAM hydrogel-based wireless bacterial detection system for dental enamel. Reproduced with permission from Ref. [174] Copyright © 2018 John Wiley & Sons Inc. **d** Wearable battery-free therapeutic diagnostic dental patches for wireless intraoral sensing and drug delivery. Reproduced with permission from Ref. [175] Copyright © 2022 Springer Nature. **e** Stretchable cocaine-sensing patch. Reproduced with permission from Ref. [176] Copyright © 2022 American Chemical Society. **f** Battery-free wireless flexible sweat sensor for multi-biomarker sensing. Reproduced with permission from Ref. [177] Copyright © 2022 The Authors-Published by American Association for the Advancement of Sciences. **g** Highly biocompatible flexible wireless wound pH-sensing patch. Reproduced with permission from Ref. [179] Copyright © 2023 John Wiley & Sons Inc. **h** Highly biocompatible flexible wireless wound pH-sensing patch for Staphylococcus aureus. Reproduced with permission from Ref. [180] Copyright © 2021 The Authors-Published by American Association for the Advancement of Sciences. **i** Commercially available dressing-based wound monitoring patch embedded with a flexible PI Cu-plated resonator. Reproduced with permission from Ref. [181] Copyright © 2021 American Chemical Society

As previously mentioned, smart contact lenses are prominent examples of noninvasive body fluid monitoring devices for wearable health assessments of tears. Park et al. designed a soft smart contact lens for sensing glucose in tears (Fig. 16a) [172]. This lens comprises transparent and stretchable nanostructured helical inductive AgNF wires and functional devices, including rectifiers, LEDs, and glucose sensors. The tear-sensing network is remotely excited through a single-loop coil connected to a VNA, which detects glucose levels and indicates them through the LED status, turning off the LED when glucose levels exceed a threshold. Furthermore, contact lenses integrating multiple detection functions can simultaneously monitor various parameters and provide comprehensive data. Kim et al. developed a wearable contact lens capable of concurrently monitoring tear glucose levels and IOP (Fig. 16b) [173]. The sensor, consisting of a helical wire inductive antenna made from a thin film of bare graphene and silver nanowires (AgNWs) with an Ecoflex membrane as a capacitor, can be modeled as an LCR circuit. Specifically, resistance varies with glucose concentration, while capacitive inductance is modulated by IOP. Both signals are wirelessly read out using a single-loop readout coil, heralding advancements in eye diagnostics.

Saliva, readily accessible at any time, reflects health status and enables early disease screening in the oral cavity. The ultra-miniaturization of passive wireless devices enables attachment to teeth without disrupting daily life. Peter et al. developed a graphene-based wireless bacterial detection system for tooth enamel (Fig. 16c) [174]. The sensing component consists of a cascade silk film/ poly(N-isopropylacrylamide) (PNIPAM) hydrogel spiral induction coil and a capacitor integrated with self-assembling peptides, which adhere to tooth enamel to detect food ingested by humans and convert it into capacitance changes, causing a resonance frequency shift. These signals can be read wirelessly via a single-loop coil, promising remote monitoring of sampling and sensing analytes as well as sensing food and liquid consumption. Additionally, wearable patches that combine sensing and drug delivery facilitate shared monitoring and treatment. Shi et al. designed a wearable, battery-free therapeutic diagnostic dental patch for wireless intraoral sensing and drug delivery (Fig. 16d) [175]. This patch includes a compact layout of Cu spiral-inductive wires, electrochemical polypyrrole/fluoride (PPy/F) on Ag/AgCl electrodes, and microcircuit modules capable of electrochemically detecting the acidic microenvironment caused by bacterial metabolism. Simultaneously, fluoride can be delivered locally from an electrically responsive drug delivery electrode for on demand treatment with wireless energy harvesting and information interaction through the designed circuitry.

Sweat, easily accessible due to the systemic distribution of sweat glands, contains electrolytes, pH, and various biomarkers indicative of physiological and pathological states. Chen et al. designed a stretchable cocaine-sensing system with sensing and energy harvesting capabilities (Fig. 16e) [176]. The sensor, comprising a Cu tape helix interconnected with an Au serpentine wire combined with thiolated anti-cocaine aptamer-modified Ag/AgCl electrodes, responds to cocaine concentrations and exhibits a frequency offset read out wirelessly using a single-loop readout coil. The concurrent detection of multiple biomarkers is becoming increasingly attractive. Liu et al. developed a battery-free, flexible wireless sweat sensor that enables the detection of multiple biomarkers, including glucose (Fig. 16f) [177]. Its passive sensing component included a Cu spiral-inductive coil and a gold serpentine interconnect connected to an aptamer-modified varactor diode. This system, wirelessly excited by a single-loop readout coil, responds to biomarker concentrations and converts potential changes to capacitance, causing a resonance frequency change that is fed back to the readout coil for interpretation.

Wound exudate analysis involves examining the fluid exuded from a wound, containing various biomarkers and microbial metabolites useful in assessing wound healing and infection risk [178]. Wireless smart sensors integrated into dressings or patches continuously monitor wound exudates and wirelessly transmit data to medical devices and smartphones. Excellent biocompatibility is crucial in such scenarios because the sensor is in direct contact with the wound. NajafiKhoshnoo et al. designed a highly biocompatible, flexible, wireless wound pH-sensing patch (Fig. 16g) [179]. This patch, consisting of an Ag spiral-inductive coil interconnected with a PANI/carbon Ag/AgCl electrode encapsulated in PDMS, can be applied to wounds to monitor pH changes indicative of healing. The patch was wirelessly read out by the designed circuitry, demonstrating its potential for remote health monitoring. Xiong et al. developed a wireless, battery-free DNA hydrogel-based wound infection Cu PI-based sensing patch to detect Staphylococcus aureus (Fig. 16h) [180]. The patch comprised a bio-responsive DNA hydrogel film, a half-wave rectified LC biosensing module, and a near field communication (NFC) module. The hydrogel binds to Staphylococcus aureus, converting its binding into capacitance changes, wirelessly transmitted to a mobile phone by the NFC module after C-V conversion. Sadaf et al. developed a wound-monitoring patch based on a commercially available dressing secondarily embedded with a flexible PI Cu-plated LC helical coil resonator (Fig. 16i) [181]. The patch responds to the extent of the wound, driven by the high dielectric constant of the wound exudate, which causes a decrease in resonance frequency. This frequency reflects the wound range and is read wirelessly using a double-loop readout coil (overlap). The availability of these wound-monitoring patches can enhance wound management, aid in site closure, and provide sterility detection.

##### Multiple Signals

Wearable patches designed for multiple-signal sensing represent groundbreaking technology with significant potential for personal health monitoring, disease diagnosis, sports science, and telemedicine. By integrating various sensors, these patches can simultaneously monitor and analyze a range of physiological signals from the human body.

**Fig. 17:**
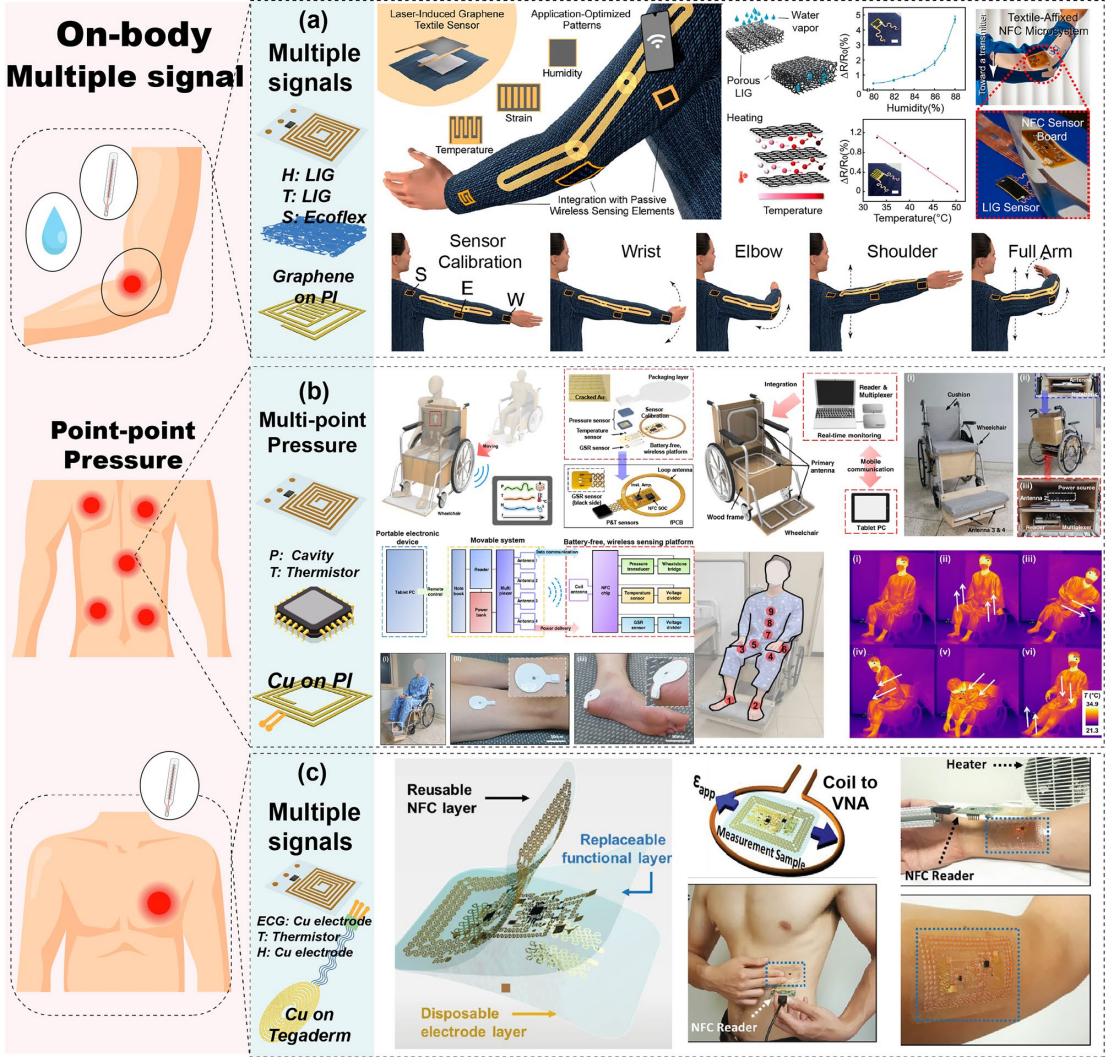
Wearable devices for multiple signals detection. **a** Multiparameter monitoring smart clothing with integrated textiles. Reproduced with permission from Ref. [182] Copyright © 2023 American Chemical Society. **b** Multipoint deployment of wireless sensing network based on human body and wheelchair. Reproduced with permission from Ref. [183] Copyright © 2021 Springer Nature. **c** Electronic tattoo for multi-sensing. Reproduced with permission from Ref. [184] Copyright © 2019 John Wiley & Sons Inc

The development of multiparameter identification and monitoring systems using integrated textiles is a current research focus. Qin et al. engineered a seamlessly coupled laser-induced graphene (LIG)-based smart garment capable of wirelessly monitoring multiple metrics (Fig. 17a) [182]. The sensing patch in this system includes a magnetic metamaterial chain as the primary network, along with multiple sensors for humidity, stress, and temperature. Humidity and pressure sensing are implemented using LIG, while strain sensing is achieved through Ecoflex. When mounted on the upper shoulder, this network can monitor diverse arm-bending postures and relay data wirelessly through an NFC hub.

Another significant area of research is the multipoint deployment of wireless sensor networks on devices such as wheelchairs, particularly beneficial for individuals suffering from conditions such as paralysis. Wu et al. devised a battery-free wireless soft-sensor network for multisite pressure and temperature measurements (Fig. 17b) [183]. This involves deploying multiple wireless patches at strategic locations across the body to provide comprehensive coverage of pressure and temperature data. The flexible wireless patch comprises a combination of Cu helical wire inductors on PI, and pressure cavity microstructure as well as temperature thermistor microcircuit modules. These patches, attachable to body regions prone to pressure damage such as the heels, ankles, knees, elbows, scapulae, and sacra, can be instrumental in minimizing pressure injuries in hospitalized or bedridden patients. Additionally, a multiplexed antenna integrated into a wheelchair precisely measures pressure and temperature distribution across the body.

Electronic tattoos, owing to their rapid deployment, untethered nature, and printability, can also be easily fabricated using multi-sensing electrode patterns. Jeong et al. developed a personalized, modular, and reconfigurable configuration for wireless multilayered electronic Cu tattoo systems (Fig. 17c) [184]. This system enables simultaneous monitoring of various physiological parameters, including electrocardiography (ECG), blood oxygen saturation (SpO_2_), heart rate (HR), skin-temperature, and skin hydration levels. The tattoo incorporates different sensing modules, each consisting of a stack of various printed passive electrodes/sensing layers paired with corresponding functional modules (such as thermistors and photodiodes), and integrates NFC technology for wireless data transmission via a single-loop readout coil. The advent of these multi-signal monitoring devices has greatly enhanced personal health management and healthcare services.

## Conclusion and Outlook

Passive wireless electronic devices offer the advantages of straightforward deployment and wireless connectivity, allowing them to perform their functions without the necessity for active components. These systems, particularly in the realm of bioelectronics and industry, hold the potential to transform current paradigms across biomedical and various other sensing applications. The impetus behind these advancements has been propelled by innovations in novel materials and processing technologies. They exhibit potential in wide applications such as harsh environments, implantable in vivo signaling, epidermal signaling, and other monitoring processes. This review comprehensively summarizes the research route from application scenarios to framework designs of passive wireless sensing systems. Harsh environments require sensors that function reliably under extreme conditions, implantable devices require biocompatibility, and wearable devices accommodate flexibility and comfort. On the basis of these requirements, the review further systematically classifies sensor modules, and readout modules, and focuses on their intricacies and interplay through various transmission modes. Sensor modules primarily pattern resonators to monitor specific samples passively, and their structures consist of single, cascade, and stent-based patterns combined with functional components (microstructure and film). Readout modules work for information interaction and energy transfer, which are composed of metal readout coils, planar antennas, and process circuits. Their interplay originates from inductive coupling. Especially, some functional modules of sensor modules require stimulation by wireless readout coils to function effectively; however, they are still classified as sensor modules because of their power-free nature. Despite numerous studies enhancing the development of passive wireless systems through optimized structural, design, and deployment strategies—thereby improving ease of configuration and facilitating seamless integration into diverse contexts—several challenges remain to be urgently addressed.

Firstly, accuracy and reliability exhibit significant challenges for sensing systems owing to their wireless transmission and interaction characteristics. The relative positions of the sensor and reader can significantly affect detection outcomes, typically necessitating fixed distances and specific alignments, which curtails the flexibility of system deployment. Potential remedies include the adoption of repeater structures that function as calibration devices providing positional data, and the integration of machine learning to furnish calibration data tailored to specific positions based on extensive frequency spectral data.

Secondly, the sensing systems' applicability to different scenarios imposes stringent requirements. Each application context demands bespoke considerations to realize practical implementations. For instance, implantable devices must maintain consistent contact with biological tissues and minimize inflammation or other adverse reactions, and the implantation process should be simplified to reduce trauma. The emergence and utilization of biodegradable materials represent a potential viable solution to these issues.

Thirdly, enhancing the sensing systems with additional technologies or functional modules could extend their capabilities beyond mere sensing. For example, integrating machine learning could diversify the applications in medical diagnosis, facilitating real-time data collection and analysis for personalized disease prediction. Moreover, real-time monitoring of industrial equipment could enable predictive maintenance schedules and preemptive alerts. Furthermore, combining this with drug delivery modules could establish a closed-loop system for simultaneous sensing and therapeutic intervention.

Future efforts should concentrate on developing long-term reliable and precisely tailored sensing solutions suitable for specific scenarios. Leveraging passive wireless technology could radically alter the landscape of personalized diagnostics and monitoring in harsh conditions, supporting robust operation in complex environments and delivering high-precision real-time data. Collectively, these wireless, battery-free sensing systems are set to become instrumental in advancing healthcare and industrial applications, representing a significant evolution in smart and autonomous monitoring technologies.

## Abbreviations

Ag: Silver
AgNWs: Silver nanowires
AgNPs: Silver nanoparticles
AgNFs: Silver nanofibers
Ag@MoS_2_: Silver nanoparticle-decorated molybdenum disulfide nanosheets
Al: Aluminum
Al_2_O_3_: Aluminum oxide
Au: Gold
AuHNWs: Hollow gold nanowires
BFC: Biofuel cell
Cr: Chromium
Cu: Copper
DOX: Doxorubicin
ECG: Electrocardiography
EEG: Electroencephalogram
EMG: Electromyography
GO: Graphene oxide
GOx: Glucose oxidase
HR: Heart rate
IAH: Ionic alginate hydrogel
ICP: Intracranial pressure
IDE: Interdigitated electrode
IOP: Intraocular pressure
IR: Intermediate relay
ITO: Tin-doped indium oxide
LC: Inductor–capacitor
LCR: Inductor–capacitor–resistor
LED: Light-emitting diode
LIG: Laser-induced graphene
LM: Liquid metal
LOD: Limit of detection
LTCC: Low-temperature co-fired ceramic
MWCNTs: Multi-walled carbon nanotubes
NFC: Near-field communication
NH_3_: Ammonia
NIR: Near infrared
OST: Oxidized starch
PANI: Polyaniline
PAPBA: Poly(3-aminophenylboronic acid)
PBA: Phenylboronic acid
PCLAU: Poly(d,l-lactide-*co*-caprolactone)
PDMS: Polydimethylsiloxane
PET: Polyethylene terephthalate
pHEMA: Poly(hydroxyethyl methacrylate)
PI: Polyimide
PLGA: Poly(lactic-*co*-glycolic acid)
PNIPAM: Poly(*N*-isopropylacrylamide)
POC: Poly(octanediol citrate)
Pt: Platinum
PtBk: Platinum black
RF: Radio frequency
RFID: Radio-frequency identification
SAW: Surface acoustic wave
SMED: Shape memory electronics
SpO_2_: Blood oxygen saturation
SRR: Split-ring resonator
Ti: Titanium
TPH: Pressure–temperature–humidity
UWB: Ultra-wideband
VNA: Vector network analyzer
WS_2_: Tungsten disulfide
